# Fecal Metabolic and Microbial Responses to Five Acute Fueling Strategies During a 60-Minute Rowing Protocol: A Randomized Crossover Multi-Omics Pilot Study

**DOI:** 10.3390/metabo16100718

**Published:** 2026-09-28

**Authors:** Xiangyu Wang, Jian Huang

**Affiliations:** Department of Physical Education, Capital Normal University, Beijing 100048, China; 7175@cnu.edu.cn

**Keywords:** microbial metabolism, personalized nutrition, carbohydrate–protein supplementation, rowing, fecal metabolomics, short-chain fatty acids, gut microbiome, crossover experiment

## Abstract

Background/Objectives: We compared next-morning fecal metabolic and microbial responses to five acute fueling strategies. Methods: Eight male recreational rowers with complete specimens from 12 crossover completers received an erythritol comparator, two carbohydrate doses, fixed carbohydrate–protein, and personalized carbohydrate–protein (P-CPS). Eighty specimens yielded 40 paired changes. Participant-blocked, period-adjusted models used restricted permutations and Benjamini–Hochberg false discovery rate (FDR) correction. Results: No primary global test passed across-modality FDR correction: SCFAs, *p* = 0.0321, q = 0.0963; metabolome, q = 0.1334; microbiome, q = 0.2813. No metabolite or originally annotated genus passed its omnibus FDR family. Post hoc SILVA 138.2 reclassification retained a nonsignificant global result but identified three low-count genus-level omnibus signals. Secondary change-score contrasts indicated higher isovaleric and isobutyric post/pre fold changes under P-CPS, fixed carbohydrate–protein, and high carbohydrate than under the comparator. However, no concentration or composite-endpoint contrast remained FDR-positive in post hoc models adjusting for same-trial pre-trial values. Caproic acid evidence also weakened with expanded correction and analytical-range checks. Two metabolome–SCFA principal-component correlations survived FDR correction and persisted after exclusion of overlapping annotations. Conclusions: This pilot identified model-sensitive secondary chemical signals without FDR-supported global intervention effects. Baseline imbalance, unmeasured beverage osmolality and fecal water, and quantification limitations preclude attributing these signals to increased fermentation or nutritional benefit.

## 1. Introduction

Carbohydrate delivery during prolonged exercise is intended to sustain carbohydrate availability and delay fatigue. Co-ingested protein may support recovery or provide additional benefit when carbohydrate delivery is suboptimal [1,2,3,4,5]. Most evidence, however, concerns group-average performance and systemic physiological outcomes. Athletes differ substantially in anthropometry, training history, dietary behavior, gastrointestinal tolerance, and metabolic response. This heterogeneity has motivated personalized nutrition approaches that use multivariable or machine-learning models to select individual strategies rather than applying a uniform dose [6,7,8,9].

The intestinal environment is a plausible but understudied source of this heterogeneity. Exercise alters splanchnic blood flow, gastrointestinal transit, substrate exposure, and host–microbial exchange. Observational comparisons link athletic status with microbial composition and metabolic capacity [10,11]. Exercise interventions can also alter fecal SCFA concentrations in a body-composition-dependent manner [12]. Acute responses are less predictable. Studies have reported fecal and circulating metabolomic changes after acute exercise, with taxonomic responses varying by exercise context [13,14]. A controlled exercise-plus-whey intervention likewise produced modest compositional and functional changes [15]. Recent work in trained rowers links training load with fecal metabolites and selected microbial features, without uniform changes in conventional diversity indices [16]. Associations also vary across sporting populations and by sex [17]. These studies do not resolve whether acute fueling strategies produce concordant chemical and taxonomic changes within the same individuals.

Fecal SCFAs require careful interpretation. Their concentrations reflect microbial formation, absorption, host utilization, intestinal transit, and sample water content rather than production alone [18,19]. Repeated sampling studies associate day-to-day fecal metabolic variation with stool moisture and pH [20]. Acetate, propionate, and butyrate are studied most often, whereas branched-chain isobutyrate and isovalerate may reflect different substrate pools. A next-morning fecal measurement integrates exercise, feeding, recovery meals, and overnight physiology. It cannot identify the producing organism or establish a beneficial response.

The parent crossover experiment compared five during-exercise strategies: an erythritol comparator, two carbohydrate doses, a fixed carbohydrate–protein strategy, and an algorithm-derived personalized carbohydrate–protein strategy (P-CPS). The recommendation framework has been described previously [6,7]. The unresolved question concerns modality-specific, within-person fecal responses to these acute exposures, rather than differences between athletes and sedentary controls. The present study does not test algorithmic prediction accuracy or assume a performance advantage for P-CPS. It examines whether the strategies yield distinguishable changes in next-morning fecal chemistry or microbial composition.

We reanalyzed matched fecal metabolomics, targeted SCFAs, and 16S rRNA gene datasets from eight participants who completed all five conditions. The primary null hypothesis was that multivariate within-trial changes did not differ among interventions within each modality. The three primary tests were evaluated together to control multiplicity across modalities. Secondary questions concerned P-CPS contrasts, sensitivity to participant influence and period adjustment, and covariance between modality-level change axes. The corresponding null hypotheses were zero adjusted differences in change and zero partial correlations. These were two-sided questions; no directional hypothesis about individual metabolites or genera was prospectively registered. The original reanalysis plan preceded examination of its results but was not registered before the parent trial. Additional post hoc analyses were not part of the original reanalysis plan. Given the small sample, high dimensionality, and single next-morning time point, all findings require a pilot interpretation.

## 2. Materials and Methods

### 2.1. Study Design and Reporting Relationship

This study was a secondary analysis of a randomized, five-condition crossover experiment. The parent study recruited 20 eligible men; eight withdrew for personal reasons and 12 completed the experiment [21]. Complete paired fecal multi-omics data were available for participants 1–8, who formed the present analysis set (Figure 1). The remaining four completers had at least one insufficient or unusable fecal specimen and lacked complete paired data across all five conditions. Each included participant completed all five conditions once, with washouts of at least 7 days. Trial order was randomized and recovered from the contemporaneous trial workbook. The analytical unit was the within-participant, within-condition change across 40 completed trials. The 80 specimens were not treated as independent participants.

The Ethics Committee of Capital University of Physical Education and Sports approved the procedures (2022A57). All participants provided written informed consent, and the study followed relevant ethical standards. The parent trial was not registered in a public trial registry. A completed CONSORT 2025 checklist with the 2019 crossover extension accompanies this report [22,23]. This multi-omics analysis shares the parent crossover experiment with earlier modeling work [7], a doctoral dissertation [21], and a separate performance-focused manuscript. The peer-reviewed modeling article treated the 60 crossover trial records as rows in a machine-learning dataset but did not report the fecal metabolomic, SCFA, or 16S outcomes analyzed here. The statistical models and multiplicity-control procedures were specified for the present multi-omics reanalysis.

### 2.2. Relationship to Earlier Reports

Three layers of overlap require distinction. First, the doctoral dissertation described the parent program and included preliminary vendor-generated analyses of these omics samples [21]. Second, the 2025 Scientific Reports article used baseline, supplement, and rowing-performance records from the same 12-person crossover cohort as 60 rows in a larger machine-learning dataset [7], but it did not analyze fecal outcomes. Third, a performance-focused manuscript from the parent experiment reports rowing and systemic physiological outcomes, not the period-adjusted fecal multi-omics analyses presented here. The current study is therefore not a new cohort or a replication of the performance experiment. It is a secondary reanalysis of a distinct outcome family in the eight participants with complete paired specimens. Its research question and inferential analyses are specific to the fecal data; descriptive rowing-distance summaries are included solely to characterize the exercise exposure (Table S16).

### 2.3. Participants

The eight participants were male university students and experienced recreational rowers. Parent-protocol eligibility required informed consent, a negative Physical Activity Readiness Questionnaire screen, and ability to complete the protocol safely. Participants reported regular endurance training (3–6 sessions/week, >1 h/session) and at least one year of rowing-ergometer experience. These were eligibility criteria, not measured distributions of training volume or experience within the subset. Severe organ dysfunction, exercise risk, or inability to participate safely were exclusion criteria. Mean (SD) age was 25.5 (2.1) years, height 180.0 (3.9) cm, and body mass 80.6 (8.6) kg. Body water was 52.9% (1.8%) and body fat was 23.5% (3.0%) (Table 1). Body composition was assessed by bioelectrical impedance using an InBody 270 analyzer (InBody Co., Ltd., Seoul, Republic of Korea). The parent protocol included preparatory-day and fasting-morning assessments; fasting and hydration conditions were not documented specifically for the baseline values summarized here. These summaries describe the eight-person subset, not the complete 12-person cohort. Quantitative habitual dietary intake and individual years of rowing experience were unavailable.

### 2.4. Exercise and Nutritional Interventions

Participants attended a familiarization trial 1 week before formal testing. During the 48 h preceding each trial, they were instructed to maintain their usual diet, activity, and sleep and to record these behaviors for review on arrival. The retained data did not permit quantitative inclusion of these records. Participants fasted after the evening meal before baseline sampling. Each trial comprised two 30 min self-paced rowing bouts at the maximal sustainable intensity on a Concept II Type D ergometer (Concept2, Morrisville, VT, USA), separated by 15 min of seated recovery. The drag factor was 120, and stroke rate was maintained at 16–24 strokes/min. Performance feedback was concealed, and standardized verbal encouragement was provided. The protocol therefore comprised 60 min of rowing within a 75 min intervention period.

Condition labels were concealed using coded beverages. Sensory matching and successful blinding could not be verified from the retained records; concealment of the labels therefore should not be interpreted as established participant blinding. The five conditions were PLA, a 900 mL beverage containing 4.5 g erythritol (0.5% *w*/*v*; 5 g/L); L-CHO, carbohydrate (The carbohydrate and protein supplements were obtained from ALL STARS Fitness Products GmbH (Peißenberg, Germany)) at 0.8 g·kg^−1^·h^−1^; and H-CHO, carbohydrate at 1.0 g·kg^−1^·h^−1^. T-CPS provided carbohydrate at 0.8 g·kg^−1^·h^−1^ plus protein at 0.2 g·kg^−1^·h^−1^. P-CPS provided individualized carbohydrate and protein according to the previously developed recommendation framework [6,7,21]. Within this subset, prescribed P-CPS carbohydrate ranged from 0.50 to 0.77 g·kg^−1^·h^−1^ (mean 0.61). Protein ranged from 0.13 to 0.19 g·kg^−1^·h^−1^ (mean 0.15), and energy from 2.52 to 3.84 kcal·kg^−1^·h^−1^ (mean 3.07). The distribution of prescriptions is summarized in Table S2; Table S6 compares all five conditions. Total beverage volume was 900 mL per condition, administered in six 150 mL portions at 0, 15, 30, 45, 60, and 75 min of the intervention. Participants consumed the protocol-specified amounts. Cumulative quantities were calculated from the reported, rounded prescription rates multiplied by 1.25 h for the 75 min protocol, including the 15 min seated rest (Table S6). Total grams equal the cumulative g/kg value multiplied by body mass in kilograms. Each 150 mL PLA portion contained 0.75 g erythritol (Shandong Sanyuan Biotechnology Co., Ltd., Binzhou, China). The investigators reported no adverse reactions during beverage administration. Osmolality was not measured for any of the five beverages. Accordingly, PLA denotes the administered comparator and does not establish physiological inertness for fecal concentration outcomes.

The recommendation model used participant characteristics and candidate supplement composition to predict rowing distance [6,7,21]. Inputs covered anthropometry and body composition, physical activity, muscular fitness, sleep, psychological questionnaires, recent smoking and alcohol use, and meal timing. Physiological inputs included blood measures, resting cardiovascular indices, heart-rate variability, and direct-current potential. Candidate carbohydrate rates were enumerated from 0.50 to 1.20 g·kg^−1^·h^−1^ in 0.01 increments, with protein approximately one-quarter of carbohydrate. The protocol selected the candidate with the highest predicted performance. The present analysis used the administered prescriptions reported in the dissertation, not a newly optimized or retrained model. P-CPS is an operational exposure label and conveys no independently established performance or microbial benefit.

### 2.5. Fecal Sampling and Sample Identity

Participants provided a fecal sample at approximately 07:30 before each trial after a fast of at least 8 h. A second sample was collected at the corresponding time the following morning. Samples were placed on dry ice and transferred to −80 °C storage within approximately 1 min. The design comprised 8 participants × 5 conditions × 2 time points = 80 samples. Sample identifiers were harmonized across metabolomics, targeted SCFAs, 16S feature tables, and paired-end sequence files before analysis. All 80 expected identifiers were present on each platform, with no unmatched participant–condition–time combination.

### 2.6. Untargeted Fecal Metabolomics

Fecal material was extracted in 600 μL methanol (Fisher Scientific, Loughborough, UK) containing 4 ppm 2-amino-3-(2-chlorophenyl)-propionic acid (Aladdin, Shanghai, China) as an internal reference. Samples were vortexed for 30 s, homogenized with steel beads at 50 Hz for 120 s, and sonicated for 10 min at room temperature. The provider reported centrifugation at 12,000 rpm for 10 min at 4 °C using an H1850-R centrifuge (Xiangyi, Changsha, China). Supernatants were passed through 0.22 μm filters (Tianjin Jinteng Experiment Equipment Co., Ltd., Tianjin, China).

Chromatography was performed on a Vanquish UHPLC system (hermo Fisher Scientific, Waltham, MA, USA) using an ACQUITY UPLC HSS T3 column (Waters Corporation, Milford, MA, USA) (150 mm length × 2.1 mm internal diameter, 1.8 μm particle size) at 40 °C, a 0.25 mL/min flow rate, and a 2-μL injection. Separate positive- and negative-ion mobile phases were used. Mass spectrometry used an Orbitrap Exploris 120 (Thermo Fisher Scientific, Waltham, MA, USA) with electrospray voltages of +3.5 kV and −2.5 kV, sheath and auxiliary gas settings of 30 and 10 arbitrary units, and a capillary temperature of 325 °C. Full-scan MS1 data were acquired from *m/z* 100–1000 at 60,000 resolution; data-dependent MS2 selected the top four ions using 30% higher-energy collisional dissociation at 15,000 resolution.

The analytical provider converted data to mzXML using ProteoWizard/MSConvert 3.0.8789 and processed peaks using XCMS centWave (https://bioconductor.org/packages/xcms/, accessed on 21 September 2026). Parameters were bandwidth 2, 15 ppm, peak width 5–30 s, *m*/*z* width 0.015, and *m*/*z* difference 0.01. The provider described annotation searches using HMDB (https://hmdb.ca/, accessed on 21 September 2026), MassBank (https://massbank.eu/MassBank/, accessed on 21 September 2026), LipidMaps (https://www.lipidmaps.org/, accessed on 21 September 2026), mzCloud (https://www.mzcloud.org/, accessed on 21 September 2026), and KEGG (https://www.kegg.jp/, accessed on 21 September 2026), with a mass-matching error <30 ppm. This annotation window exceeds both the 15 ppm peak-detection tolerance and the manufacturer’s routine external-calibration mass-accuracy specification of <3 ppm under defined conditions [24]. The broader matching window increases annotation ambiguity; it is a processing threshold, not a measurement of the mass accuracy achieved in this sample batch. Provider quality control included LOESS signal correction and exclusion of features with a QC relative standard deviation >30%. This threshold was passed by 85.8% of positive-mode and 83.1% of negative-mode features. The available abundance matrix contained 1051 provisionally annotated features, comprising 720 positive-mode and 331 negative-mode features, across all 80 samples. Raw acquisition data were unavailable, so peak detection and provider correction could not be repeated independently [25,26,27].

The provider’s “level 2” field was not accepted as a verified MSI confidence assignment. Every matrix entry linked to a provider-exported identification plot, and the inspected examples displayed experimental/reference mirror spectra. However, the tables lacked feature-specific MS2 acquisition flags, scan mappings, and numerical spectral-match scores; a verified acquired-MS2 coverage count could not be established from the retained records. The library field included 137 MetaDNA annotations, which were not treated as equivalent to independently verified direct library matches. We therefore retain names as provisional candidate labels rather than claiming MSI level 2 for the complete matrix. Without documented evidence supporting a specific identity, interpretation is restricted to MSI level 3 compound classes where class-level evidence is available, or to unidentified features (MSI level 4) otherwise [25]. Table S18 summarizes the retained annotation evidence and mass-error audit.

Feature values were log_2_ transformed and each sample was median centered separately within positive and negative ionization modes. No missing or nonpositive study values required imputation. Next-morning minus pre-trial changes were then calculated for each participant and condition. All 1051 features had nonzero change variance and entered the analysis without outcome-based screening. Feature identifiers were qualified by ionization mode where necessary to distinguish two different annotations sharing one provider identifier. Unsupervised principal-component analysis (PCA) summarized standardized feature changes.

### 2.7. Targeted Fecal SCFA Quantification

Seven acids (obtained from Sigma-Aldrich, Shanghai, China) were quantified: acetic, propionic (Sinopharm, Shanghai, China), isobutyric, butyric, isovaleric, valeric, and caproic acids. Fecal material was homogenized with water and grinding beads and centrifuged. An aliquot of 200 μL supernatant was combined with 100 μL 15% phosphoric acid, 20 μL 375 μg/mL 4-methylvaleric acid (Sigma-Aldrich, Shanghai, China), and 280 μL ether (Titan, Shanghai, China). The mixture was vortexed for 1 min and centrifuged. The provider described centrifugation at 12,000 rpm for 10 min at 4 °C on an H1850-R instrument. Analysis used a Thermo TRACE 1300 gas chromatograph (Thermo Fisher Scientific, Waltham, MA, USA) coupled to an ISQ 7000 mass spectrometer (Thermo Fisher Scientific, Waltham, MA, USA) and an HP-INNOWAX column (Agilent Technologies, Santa Clara, CA, USA) (30 m × 0.25 mm × 0.25 μm 30 m length × 0.25 mm internal diameter, 0.25 μm film thickness). Helium flow was 1 mL/min; 1 μL was injected at a 10:1 split. Inlet, ion-source, and transfer-line temperatures were 250 °C, 300 °C, and 250 °C, respectively. The oven increased from 90 °C to 120 °C at 10 °C/min, to 150 °C at 5 °C/min, and to 250 °C at 25 °C/min. The final temperature was held for 2 min. Electron ionization at 70 eV and selected-ion monitoring were used. The results were supplied as extract concentrations (μg/mL) and fecal contents (μg/g). Additional centrifugation and calibration details are provided in the Supplementary Methods.

Both retained calibration tables reported a limit of quantification (LOQ) of 0.02 μg/mL for each acid. These thresholds apply to extract concentrations, not directly to fecal μg/g values. We checked the provider’s conversion against sample mass and extraction factors. Measurements at or below LOQ and at or below twice LOQ were flagged separately; the latter defined proximity to LOQ. The two calibration tables shared this LOQ but could not be assigned to individual acquisition batches. Most upper calibration limits were 500 μg/mL; caproic acid had an upper limit of 250 μg/mL in one table. Original positive values were retained for comparability, with low and out-of-range measurements explicitly identified.

All 80 samples had positive provider-reported values for all seven analytes, although positivity did not imply reliable quantification above LOQ. Concentrations were log_2_ transformed, and next-morning minus pre-trial changes were calculated. PCA and a standardized seven-analyte global test summarized the profile; analyte-specific models were secondary. Absolute concentration summaries by condition and time point and analytical-range counts are supplied in Tables S7 and S8. Complete aggregate statistical results and analysis code are supplied in Supplementary Data S1; individual source values remain subject to the Data Availability Statement.

### 2.8. 16S rRNA Gene Amplicon Data

The supplied dataset contained paired-end Illumina (San Diego, CA, USA) FASTQ files for every specimen and a complete taxonomy-associated feature table. According to the analytical report, reads were primer-trimmed with cutadapt (https://cutadapt.readthedocs.io/en/stable/, accessed on 21 September 2026) and processed in QIIME 2 (version 2019.4). DADA2 (https://benjjneb.github.io/dada2/, accessed on 21 September 2026) was used for quality filtering, denoising, paired-read joining, and chimera removal, after which singleton amplicon sequence variants (ASVs) were removed [28,29]. The full table contained 35,735 ASVs. Rarefaction to 30,357 reads retained 34,689 ASVs across the 80 samples. The imported taxonomy artifact lacked classifier provenance. The provider report names Greengenes release 13.8 as its default, and the delivered bracketed family names and placeholder genera are consistent with Greengenes 13_8; we therefore identify this as the probable original source, without claiming recovery of the exact classifier. Read-end inspection supported the 338F (ACTCCTACGGGAGGCAGCAG) and 806R (GGACTACHVGGGTWTCTAAT) primer pair targeting V3–V4. This is a sequence-based reconstruction, not a recovered laboratory primer record (Supplementary Methods S1.9).

Alpha-diversity endpoints were observed features, Shannon index, Simpson index, Chao1, and Good’s coverage. ASV-level Bray–Curtis and Jaccard distances described the magnitude of change between paired pre-trial and next-morning samples. The original genus CLR analyses used the unrarefied count table; rarefaction was not applied to that matrix. Chloroplast- and mitochondrial-labeled features were removed before aggregation. The provider’s fallback labels, including unresolved higher-rank placeholders, were retained when genus assignment was unavailable. Genera present in at least 20% of samples and with mean relative abundance ≥0.01% were retained (88 of 382 aggregate labels). A 0.5-count pseudocount was added before centered log-ratio (CLR) transformation [30]. Next-morning minus pre-trial CLR changes were analyzed at the genus level. Dispersion was tested separately from centroid effects. The complete filtering audit and per-label read-count summaries are supplied in Table S11 and Supplementary Data S1.

To address dependence on the original taxonomy, we reclassified all 35,735 retained representative ASVs using the official SILVA 138.2 SSU Ref NR99 full-length, uniform-prior classifier for QIIME 2 release 2025.7 [31,32,33]. Classification used scikit-learn 1.4.2 and the q2-feature-classifier (version 2025.7.0) prediction and hierarchical confidence rules at a threshold of 0.7, with automatic orientation. The exported model was run through a Python input/output adapter; the reference model and prediction rules were unchanged. Denoising and the ASV count table were retained. After organelle removal, counts were grouped by the full lineage through genus, retaining unresolved assignments at their deepest classified rank. This avoided combining identically named unresolved categories across different families. Applying the same prevalence and mean-abundance thresholds retained 130 of 507 taxonomic labels, including 24 unresolved categories. CLR transformation again used a 0.5-count pseudocount. This post hoc sensitivity changes the reference, classifier, and aggregation key together; it is not a database-only comparison or an independent replication.

### 2.9. Statistical Analysis

The statistical analysis plan was finalized before the present reanalysis. For each outcome, the primary response was within-trial change: next-morning minus pre-trial. Participant and trial period were retained irrespective of significance. The blocked linear model was as follows:

change~participant + trial period + intervention,

where participant and the five-level trial period were categorical fixed effects. The intervention term was tested against the nested model without intervention. Feature-level estimates are adjusted mean differences in change. Four P-CPS comparisons (versus PLA, L-CHO, T-CPS, and H-CHO) were specified before examination of the original reanalysis results. They were not preregistered before the parent crossover trial and remain secondary. For log_2_ SCFA outcomes, exponentiating an estimate and its confidence limits gives the ratio of condition-specific post/pre fold changes. This quantity is not the ratio of next-morning absolute concentrations. Confidence intervals are unadjusted two-sided 95% intervals.

For each modality, a global multivariate trace pseudo-F statistic compared residual sums of squares from reduced and full models across the complete change matrix. Metabolite and SCFA variables were standardized before this test; genus CLR values were already on comparable log-ratio scales. Significance was obtained from 9999 Freedman–Lane permutations of reduced-model residuals, restricted within participant [34,35]. The random seed was 20260806. Partial R^2^ represented the proportional reduction in residual sum of squares attributable to intervention. The median and 95th percentile of its permutation-null distribution contextualized the observed value. Partial R^2^ was treated as a sample-specific descriptive measure, not a stable population estimate. Benjamini–Hochberg adjustment was applied jointly to the three primary global *p* values [36].

Pairwise contrasts used two-sided tests. FDR was controlled separately for the original secondary families [36]: 1051 metabolite omnibus tests, 88 genus omnibus tests, five alpha-diversity tests, and seven SCFA omnibus tests. Each original P-CPS contrast was adjusted across all features in its modality. The decision threshold was q < 0.05. Nominal *p* < 0.05 without the applicable multiplicity correction was not treated as confirmatory evidence or used to select mechanistic pathways. The four-active-condition-versus-PLA analysis was post hoc. We report both seven-analyte within-contrast q values and a joint family of 28 contrasts. Interpretation of that expanded analysis uses the 28-comparison adjustment.

Original sensitivity analyses omitted trial period, removed one participant at a time, and used exact paired Wilcoxon tests for key P-CPS contrasts. Cross-omics integration used the first three unsupervised change PCs from each modality. Partial Spearman correlations were obtained by ranking PC scores and residualizing participant and period. Restricted within-participant permutations (4999) generated *p* values, with all 27 PC correlations FDR-adjusted together. Intervention was not additionally residualized, so associations can include shared intervention-related variation. Feature-level correlations selected from the same dataset remain exploratory and are not used for mechanistic claims. Analyses were implemented in Python 3.12; scripts, dependency versions, model definitions, input schemas, and aggregate results are supplied in Supplementary Data S1. The scripts require the restricted participant-level inputs for independent execution. Generative AI assisted with code development and checking; specific tasks and product information are disclosed in the Acknowledgments.

Additional post hoc SCFA analyses evaluated natural-log CLR changes in the seven-acid mass composition, total SCFA content, branched-chain mass percentage, and a branched-to-major-straight-chain log_2_ ratio. Branched-chain acids were isobutyrate plus isovalerate; the straight-chain denominator comprised acetate, propionate, and butyrate. Mass percentages were not described as molar fractions. FDR families comprised seven CLR omnibus tests, 28 CLR contrasts, three composite-endpoint omnibus tests, and 12 composite-endpoint contrasts. A separate restricted permutation test summarized the CLR profile. These exploratory families were not promoted to primary outcomes. Analytical-range sensitivity omitted acids with any value at or below LOQ. The Supplementary Methods specify every transformation and family.

Annotation-based cross-omics sensitivity analyses excluded the two directly matched SCFA features and, separately, a broader chemically defined set of related features. Exclusion rules were fixed before computing the corresponding sensitivity results. Mode-specific centering and PCA were repeated after each exclusion, followed by all 27 PC correlations and their FDR correction. A separate post hoc microbiome analysis removed Pseudomonas and Acinetobacter and reconstructed CLR profiles from the retained counts. This assesses numerical sensitivity to those labels; it cannot diagnose laboratory contamination.

A further post hoc sensitivity test assessed dependence on pre-trial values. For each SCFA, log_2_ next-morning concentration was modeled with participant, categorical period, intervention, and the same-trial log_2_ pre-trial concentration as predictors. The three previously defined composite endpoints were analyzed analogously on their existing scales. Each endpoint had a common baseline slope across conditions, 40 trials, and 23 residual degrees of freedom. BH adjustment covered all 28 acid contrasts and, separately, all 12 composite contrasts; omnibus tests used separate seven-acid and three-composite families. Exponentiated coefficients in these models are baseline-adjusted next-morning ratios, not ratios of post/pre fold changes. These sensitivity models did not replace the original change-score analysis (Table S14).

For the SILVA sensitivity, the same participant-blocked, categorical-period-adjusted models and 9999 restricted permutations were repeated. BH adjustment covered all 130 label-specific omnibus tests and, separately, all 130 tests in each of the four P-CPS-versus-comparator contrast families. A separately labeled alternative three-test global family substituted the SILVA microbiome *p* value for the original microbiome *p* value while retaining the metabolome and SCFA *p* values. The original primary family and original annotation-based PCA and cross-omics analyses were retained alongside this sensitivity (Table S17).

## 3. Results

### 3.1. Data Completeness and Participant Characteristics

All eight participants completed all five trial conditions, yielding 40 paired trial changes. Metabolomics, SCFAs, the full and rarefied ASV tables, and paired-end sequencing files each contained the expected 80 specimens. There were no cross-platform identifier mismatches. The range of participant age was 23–30 years, height 173–185 cm, and body mass 66.2–89.0 kg (Table 1). Mean completed distance across the two rowing bouts ranged from 10,415.8 m under PLA to 12,150.3 m under P-CPS in this eight-person subset. Condition-specific mean (SD) values for this subset and the 12-person parent cohort are reported separately in Table S16. These descriptive summaries characterize the exercise exposure and do not constitute a new performance-efficacy analysis.

### 3.2. Global Tests Did Not Meet the Across-Modality FDR Threshold

None of the three primary global tests met q < 0.05 after correction across modalities (Table 2). The seven-SCFA profile had a nominal permutation *p* value of 0.0321 (pseudo-F_4,24_ = 1.935), but q = 0.0963. The metabolome yielded *p* = 0.0889 and q = 0.1334; the genus CLR profile yielded *p* = 0.2813 and q = 0.2813. The observed partial R^2^ values were 0.244, 0.160, and 0.151 for SCFAs, metabolome, and microbiome, respectively. Their corresponding null medians were 0.128, 0.141, and 0.141, and null 95th percentiles were 0.229, 0.165, and 0.170. The nonzero null values illustrate the contribution of model flexibility in this small sample. Descriptive PCA plots do not alter the global inference (Figure 2). Metabolome PC1 and PC2 explained only 12.0% and 7.0% of variance; corresponding values were 56.4% and 20.7% for SCFAs and 17.5% and 8.5% for genus CLR profiles.

### 3.3. Metabolite Features Did Not Survive Multiplicity Correction

Sixty-six of 1051 putatively annotated metabolite features had nominal omnibus *p* < 0.05, but none passed FDR correction at q < 0.05 or q < 0.10. No metabolite passed FDR correction in any of the four original P-CPS contrast families. We therefore did not use nominal features for pathway enrichment or directional biological claims. The metabolomic findings did not establish a robust global or feature-level intervention signature.

### 3.4. Secondary SCFA Contrasts and Compositional Sensitivity

Isovaleric, isobutyric, and caproic acids showed nominal analyte-level omnibus effects (*p* = 0.0118, 0.0227, and 0.0312), but all had q = 0.0728. No acid passed the seven-analyte omnibus family. Complete absolute concentration summaries and aggregate plots are provided in Table S7 and Figure S1. All seven P-CPS-versus-PLA log_2_-change estimates were positive (Table 3); this common direction requires consideration of concentration-scale and comparator effects.

Within the original seven-acid P-CPS-versus-PLA secondary family, isovaleric and isobutyric acid changes were higher under P-CPS (q = 0.0178 and 0.0182). The corresponding ratios of post/pre fold changes were 8.62 (95% CI 2.30–32.24) and 6.04 (1.81–20.23). Caproic acid had a ratio of 4.13 (1.29–13.27; q = 0.0451). These are between-condition ratios of within-condition fold changes, not ratios of post-trial concentrations. Their wide, unadjusted intervals show considerable uncertainty, and selection of large secondary estimates may inflate apparent effects in a pilot sample [37]. Directions were unchanged in all eight leave-one-participant-out analyses. Exact paired Wilcoxon q values were 0.0273 for both branched-chain acids and 0.0547 for caproic acid (Table S4). No SCFA passed the original P-CPS-versus-active-comparator FDR families; all 21 such comparisons, including log_2_ estimates and ratios of fold changes, are reported in Table S15.

The complete post hoc analysis of four active conditions versus PLA comprised 28 contrasts (Figure 3; Table S9). Isovaleric and isobutyric acid fold-change ratios were higher under T-CPS, H-CHO, and P-CPS after correction across all 28 comparisons (q = 0.0334–0.0400). No L-CHO contrast passed this correction. P-CPS caproic acid no longer passed it (q = 0.0677). Thus, the branched-chain concentration pattern was not unique to P-CPS.

Analytical-range checks identified 8/80 caproic acid estimates at or below LOQ and 31/80 at or below twice LOQ, including the eight lower values. One valeric acid estimate was at or below LOQ. None of the remaining five acids had estimates at or below twice LOQ. One next-morning acetate estimate under H-CHO was 519.2115 μg/mL, exceeding the listed 500 μg/mL upper calibration limit; sample-specific dilution or repeat-analysis documentation was unavailable (Table S8). Original values were retained in the analyses, with these analytical-range limitations explicitly reported.

The exploratory seven-acid CLR composition test yielded pseudo-F_4,24_ = 2.195, *p* = 0.0199, and partial R^2^ = 0.268. No CLR analyte omnibus test passed FDR; only the T-CPS-versus-PLA propionate contrast passed correction across 28 CLR contrasts (q = 0.0437). The omnibus test for total measured SCFA content was not significant (*p* = q = 0.2283). Branched-chain mass percentage and the branched-to-major-straight-chain log ratio each had omnibus q = 0.0437. Across 12 composite contrasts, T-CPS showed a greater change in branched-chain mass percentage than PLA (4.61 percentage points, 95% CI 1.74–7.47; q = 0.0282). The branched-to-major-straight-chain ratio of fold changes was 6.27 for T-CPS versus PLA (1.86–21.13; q = 0.0282) and 5.43 for P-CPS versus PLA (1.56–18.90; q = 0.0399). The P-CPS mass-percentage contrast did not pass this correction (q = 0.0663) (Table S10; Figure S2).

Excluding caproic and valeric acids because of their at-or-below-LOQ observations yielded a five-acid concentration-profile *p* = 0.0713 and CLR-profile *p* = 0.0296. The respective q values across these two post hoc sensitivity tests were 0.0713 and 0.0592. These findings depend on the analytical scale and included acids. Relative redistribution is possible, but these checks do not establish independence from fecal-matrix effects or analytical uncertainty.

Pre-trial branched-chain concentrations differed descriptively across conditions. Mean isovaleric and isobutyric acid concentrations were 89.29 and 87.91 μg/g under PLA, compared with 26.97 and 33.93 μg/g under P-CPS and 22.27 and 29.15 μg/g under T-CPS (Table S7). In the post hoc baseline-adjusted models, no acid contrast passed BH across 28 tests (all q ≥ 0.3682), and no composite contrast passed BH across 12 tests (all q ≥ 0.2614). For P-CPS versus PLA, baseline-adjusted next-morning ratios were 2.22 (95% CI 0.82–6.02) for isovaleric acid and 2.14 (0.78–5.92) for isobutyric acid; these are different estimands from the original ratios of fold changes. The adjusted branched-to-major-straight-chain ratio contrast also remained nonsignificant (q = 0.2614). All estimates, including negative findings, are provided in Table S14. The secondary SCFA findings were therefore sensitive to the handling of pre-trial concentration.

### 3.5. Microbiome Diversity and Composition

None of the five alpha-diversity changes differed across interventions after FDR correction (all q ≥ 0.687; Table S3). The magnitude of pre-trial-to-next-morning change did not differ by intervention for Bray–Curtis distance (*p* = 0.850) or period-adjusted Jaccard distance (*p* = 0.109). The Jaccard result was nominal when period was omitted (*p* = 0.029), demonstrating sensitivity to crossover period. Multivariate dispersion did not differ across interventions (*p* = 0.562).

Under the original provider annotations, six of 88 genus-level omnibus tests had nominal *p* < 0.05, but none passed FDR correction. Lactobacillus had the lowest omnibus *p* value (*p* = 0.000983, q = 0.0865). The original P-CPS-versus-T-CPS contrast family identified lower CLR changes for Lactobacillus, Pseudomonas, Acinetobacter, and Allobaculum. Lactobacillus also passed the P-CPS-versus-H-CHO contrast-specific adjustment. Full estimates and sensitivity results are retained in Supplementary Data S1. No genus passed FDR correction for P-CPS versus PLA or L-CHO. These isolated secondary signals do not establish broad community restructuring. Pseudomonas and Acinetobacter are also recognized potential reagent-associated taxa [38]. No mechanistic interpretation is assigned to these two low-count labels. Their source cannot be resolved without suitable extraction and reagent controls.

Across 88 retained genus-level labels, the median of the per-label median read counts was 10.25 (range of medians, 0–3476); 26 labels had a median of three reads or fewer. Zero-count samples were included. Pseudomonas and Acinetobacter had median counts of 11 and 9, respectively. Removing both labels and recomputing CLR left 86 labels and yielded a post hoc global pseudo-F_4,24_ = 0.992, *p* = 0.4856, and partial R^2^ = 0.142 (Table S11). This sensitivity analysis likewise did not provide global evidence of an intervention effect and cannot determine whether either label reflects contamination.

With SILVA 138.2 classification, the 130-label global test remained nonsignificant (pseudo-F_4,24_ = 1.110, *p* = 0.1932, partial R^2^ = 0.156; alternative three-test q = 0.1932). However, three label-level omnibus tests passed BH correction: Lactiplantibacillus (q = 0.0000374), Pseudomonas (q = 0.00842), and Lactobacillus (q = 0.0207). Their median counts across all 80 samples, including zeros, were zero, 12, and nine reads, respectively. The four P-CPS contrast families yielded two, two, three, and two FDR-positive comparisons versus PLA, L-CHO, T-CPS, and H-CHO, respectively (Table S17). Thus, the absence of global evidence was stable across these taxonomic analyses, whereas the individual-label findings were not. Complete results for all 130 omnibus tests and all 520 contrasts are supplied in Supplementary Data S1.

### 3.6. Cross-Omics Covariance

Two of the 27 original-analysis partial Spearman correlations survived FDR correction (Figure 4; Table S5): metabolome PC1 with SCFA PC1 (ρ = 0.653, permutation *p* = 0.0002, q = 0.0054) and metabolome PC2 with SCFA PC2 (ρ = 0.538, *p* = 0.0014, q = 0.0189). No microbiome PC correlation survived correction. Metabolome PC1 and PC2 together explained 18.95% of change variance, so these associations describe selected axes, not the complete metabolome. Excluding the two directly overlapping SCFA annotations retained the corresponding correlations (ρ = 0.659 and 0.541); excluding a broader set of 12 related annotations also retained them (ρ = 0.657 and 0.542). The respective q values remained 0.0054 and 0.0189 in each scenario (Tables S12 and S13). Mode-specific centering and all 27 tests were repeated within each scenario. These checks reduce concern about direct feature duplication but do not establish independent biological validation.

## 4. Discussion

The main inference is that none of the three modality-wide intervention tests passed the joint primary FDR correction. The SCFA profile had a nominal signal, whereas metabolome and microbiome global *p* values were 0.0889 and 0.2813. Secondary change-score analyses suggested chemical differences relative to the erythritol comparator, but these concentration and composite findings did not remain FDR-positive after same-trial baseline adjustment. No global taxonomic difference passed FDR correction with either the original annotations or SILVA 138.2. The latter nevertheless identified three low-count omnibus signals, demonstrating the sensitivity of individual-label conclusions to taxonomic processing. Two metabolome–SCFA PC associations persisted after annotation-based exclusion. Together, these results identify hypotheses for replication without establishing a global treatment effect, increased microbial fermentation, or a benefit of personalized fueling.

The distinction between global and secondary findings is essential. The original nominal SCFA global result becomes q = 0.0963 when all three primary tests are considered together. Partial R^2^ values should also be read against their nonzero permutation-null distributions, especially with only eight participants. The apparent P-CPS-versus-PLA branched-chain differences persisted in the expanded contrast family, but similar differences appeared under T-CPS and H-CHO. Caproic acid was less stable across multiplicity definitions, exact tests, and analytical-range checks. Large fold-change ratios with wide intervals can arise when comparator changes are small or variable. Their magnitudes should not be adopted as reliable population effects or used directly for trial planning [37,39]. The high pre-trial branched-chain concentrations under PLA, their subsequent decline, and the attenuation after adjustment for same-trial pre-trial values support baseline imbalance and regression to the mean as a parsimonious explanation for the large change-score contrasts (Tables S7 and S14). Thus, the contrast of a fall from a high comparator baseline with increases from lower active-condition baselines should not be interpreted as evidence of greater nutrient-induced fermentation. The baseline-adjusted models are post hoc, assume a common endpoint-specific baseline slope, and remain imprecise in eight participants. They cannot quantify the contribution of regression to the mean separately from other sources of variation or prove the absence of an intervention effect. The lack of agreement across baseline-handling methods limits the biological interpretation of the secondary findings.

Fecal concentrations cannot establish intestinal production. Most SCFAs are absorbed, and stool content depends on transit, water content, epithelial uptake, and host metabolism [18,19,20]. Branched-chain isobutyrate and isovalerate are associated with branched-chain amino-acid fermentation. Experiments with human fecal microbiota show that protein source can influence short-chain and branched-chain acid production [40]. These in vitro findings do not establish the substrate source or health significance of our next-morning measurements. Recovery diets, transit, dry mass, microbial function, flux, and absorption were not quantified. The observed concentration and compositional contrasts therefore cannot be translated into increased proteolytic fermentation or a beneficial nutritional response.

The erythritol comparator requires a dose-specific interpretation. PLA contained 4.5 g in 900 mL (0.5% *w*/*v*), divided into six 0.75 g portions. Human dose-ranging studies show dose-dependent, saturable absorption [41]. A human fecal microbiota experiment found no detectable erythritol fermentation over 24 h [42], and a regulatory reassessment judged intestinal microbial metabolism negligible [43]. Storey et al. administered 20, 35, or 50 g erythritol in 400 mL liquid; the 20 and 35 g doses did not significantly increase reported gastrointestinal symptoms relative to the control, whereas 50 g increased nausea and borborygmi [44]. Our total dose was lower and divided into six portions, but their experiment did not involve exercise. This literature provides context for the low comparator dose but does not establish its neutrality during prolonged exercise. The investigators reported no adverse reactions during administration; this observation does not exclude subclinical changes in intestinal water handling or fecal concentration. Osmolality was not measured for any beverage, and stool water was not quantified. Consequently, the contribution of these factors cannot be estimated. A nutrient-versus-PLA contrast cannot separate nutrient effects from erythritol-comparator effects. Ratio-based analyses remove a uniform within-sample scaling factor, but cannot recover stool water or rule out nonuniform analytical and physiological effects.

The fixed beverages provided two planned comparator pairs: T-CPS and L-CHO supplied the same carbohydrate rate (0.8 g·kg^−1^·h^−1^), whereas T-CPS and H-CHO supplied the same carbohydrate-plus-protein energy rate (4.0 kcal·kg^−1^·h^−1^) with different nutrient compositions [21]. These comparisons distinguish matching on carbohydrate from matching on energy. In the present subset, every P-CPS carbohydrate prescription was below both fixed carbohydrate doses, and mean P-CPS energy delivery was the lowest among the active conditions. P-CPS contrasts therefore compare complete fueling strategies and cannot isolate personalization or any single nutrient. The extended comparisons showed branched-chain concentration differences relative to PLA under T-CPS and H-CHO as well as P-CPS. Because H-CHO supplied no protein, this pattern does not support a simple protein-dose explanation. No original P-CPS-versus-active-comparator SCFA contrast passed its FDR family.

The absence of a broad 16S response over this time scale is biologically plausible. Taxonomic composition can remain comparatively stable over hours to one day even when the chemical environment changes. Furthermore, 16S relative abundance does not measure transcription, enzyme activity, or absolute microbial load. Acute exercise studies have reported metabolomic responses, with taxonomic changes varying by exercise context [13,14]. Controlled exercise interventions have produced modest compositional and functional changes [15]. However, studies of professional, elite, or highly trained athletes [10,11,16] are not directly interchangeable with this recreational university cohort. Microbial associations vary across sports and by sex [17], limiting extrapolation from eight men. Our results do not establish microbial inactivity; the measured genus-level composition did not provide FDR-robust global evidence of a differential response.

The genus-level findings warrant particular caution. No label passed the original 88-label omnibus FDR family, whereas three passed the 130-label family after SILVA 138.2 reclassification. This difference prevents a general claim that every taxon-level analysis was negative. The retained global result does not make the new feature-level signals confirmatory. CLR estimates describe relative log ratios, not absolute cell numbers. Low counts increase sensitivity to pseudocounts, stochastic sampling, and laboratory background [30,38]. Differential-abundance findings can also vary with preprocessing and analytical methods [45]. The updated reference and classifier split or regrouped original labels, including Lactobacillus, and the lineage-based aggregation rule changed unresolved categories. Numerical differences therefore cannot be attributed solely to nomenclature updates. Pseudomonas and Acinetobacter may represent biological signal, background contamination, or both; their occurrence alone cannot distinguish these explanations. Removal sensitivity does not substitute for negative controls. Neither the original nor updated low-count signals establish microbial benefit or a fermentation mechanism. They require independent validation with appropriate controls and absolute or targeted quantitative assays.

The cross-omics results support covariance between selected chemical axes rather than a demonstrated microbial mechanism. Integration findings depend on feature representation and analytical method [46]. Here, the leading two metabolomic PCs captured approximately 19% of total change variance, and the genus-level axes did not show FDR-supported correlations. Removing direct and broader SCFA-related annotations left the two metabolome–SCFA associations intact. This argues against their being explained solely by those duplicated annotations, but both platforms still sampled the same fecal matrix. Shared water content, transit, recovery diet, or analytical effects remain plausible contributors. Participant and period adjustment did not additionally remove intervention-related variation. The associations therefore cannot identify a producing organism, establish mediation, or serve as independent replication.

The present analysis does not depend on findings from the performance-focused report. P-CPS was defined by a recommendation framework documented in the parent research program and related modeling studies [6,7,21]. Whether that strategy improves rowing performance is not a premise of this fecal analysis. The current data also do not test algorithm accuracy or identify an optimal supplement. Accordingly, the multi-omics observations are not offered as mechanistic explanations for a performance claim. Completed distance was not matched across the self-paced conditions (Table S16), which further limits attributing fecal contrasts to beverage composition alone.

This reanalysis also differs from the preliminary dissertation chapter [21]. That chapter used vendor-generated OPLS-DA, nominal *p* and variable-importance thresholds for pairwise metabolite screening, and within-condition pre/post tests for selected microbial diversity indices. The current analysis instead treats participant and categorical period as design factors, begins with global intervention tests of change, and controls FDR within defined outcome families. The earlier pathway lists and isolated pre/post diversity findings are therefore not reproduced as confirmatory results. These differences arise from the estimands and multiplicity control, not from different specimens.

The study’s main strength is its matched, repeated design across three measurement platforms. Every included participant contributed all five interventions and both sampling times, and participant blocking avoided pseudoreplication of the 80 specimens. Trial period was modeled explicitly. Complete statistical families, negative findings, concentration-scale summaries, and sensitivity results are reported together. Reanalysis of retained inputs reproduced the original key estimates, and the new analyses provide additional checks on their interpretation. Complete-case availability, however, does not remove potential selection bias from the four parent-study completers outside the omics analysis.

This pilot has several limitations. Sensory matching and a blinding check were not documented, so differences in perceived beverage characteristics could have influenced expectations or self-paced effort. Eight men provide limited precision and external validity; no prospective sample-size calculation was performed for these secondary omics outcomes. Effects highlighted after statistical screening may be inflated, especially in a small discovery dataset [37]. One next-morning specimen integrates exercise, supplementation, recovery intake, transit, and overnight physiology, without resolving their temporal contributions. Although participants were asked to record their usual diet, activity, and sleep, quantitative logs and recovery meals were unavailable for adjustment. Stool consistency, dry mass, quantitative gastrointestinal symptom scores, and recent antibiotic exposure were also unavailable. Although the comparator contained 4.5 g erythritol and the investigators reported no adverse reactions, beverage osmolality and fecal water were not measured. Clinical tolerability therefore cannot establish neutrality for fecal concentration outcomes. Analytical-range limitations particularly weaken caproic acid interpretation. Non-targeted raw spectra and authentic-standard confirmation were unavailable, so the provisional identities and upstream peak processing could not be independently validated; the broad <30 ppm annotation window adds uncertainty [25,27]. The original 16S classifier provenance and laboratory-control information were incomplete. SILVA 138.2 reclassification addresses the taxonomic limitation but cannot recover missing controls or measure absolute microbial load. The secondary comparisons were not preregistered before sample collection; additional analyses are post hoc and cannot provide independent confirmation.

Future studies should specify a small set of primary outcomes and a multiplicity plan before recruitment. Sample size should reflect a scientifically meaningful effect or desired precision, rather than the largest estimate observed in this pilot [39]. Larger, sex-diverse cohorts should include intermediate recovery samples alongside next-day specimens. Comparator formulations should be specified and beverage osmolality measured, with standardized recovery meals and gastrointestinal monitoring. Stool water or dry-mass normalization, analytical-range verification, absolute microbial quantification, and functional measurements would help separate concentration effects from metabolism. Replication of branched-chain composition and chemical covariance should precede claims about personalization, fermentation mechanisms, or host benefit.

## 5. Conclusions

The three primary modality-wide tests did not meet the across-modality FDR threshold in this eight-person crossover pilot. Secondary SCFA concentration and compositional signals were comparator-dependent and sensitive to adjustment for pre-trial values. Metabolome–SCFA covariance persisted after annotation exclusions, but no FDR-supported global taxonomic response was detected. Taxon-level signals changed after contemporary reclassification and remain exploratory. These findings do not establish increased fermentation or nutritional benefit. Baseline imbalance, unmeasured beverage osmolality and fecal water, analytical-range limitations, and small-sample imprecision require independent replication.

## Figures and Tables

**Figure 1:**
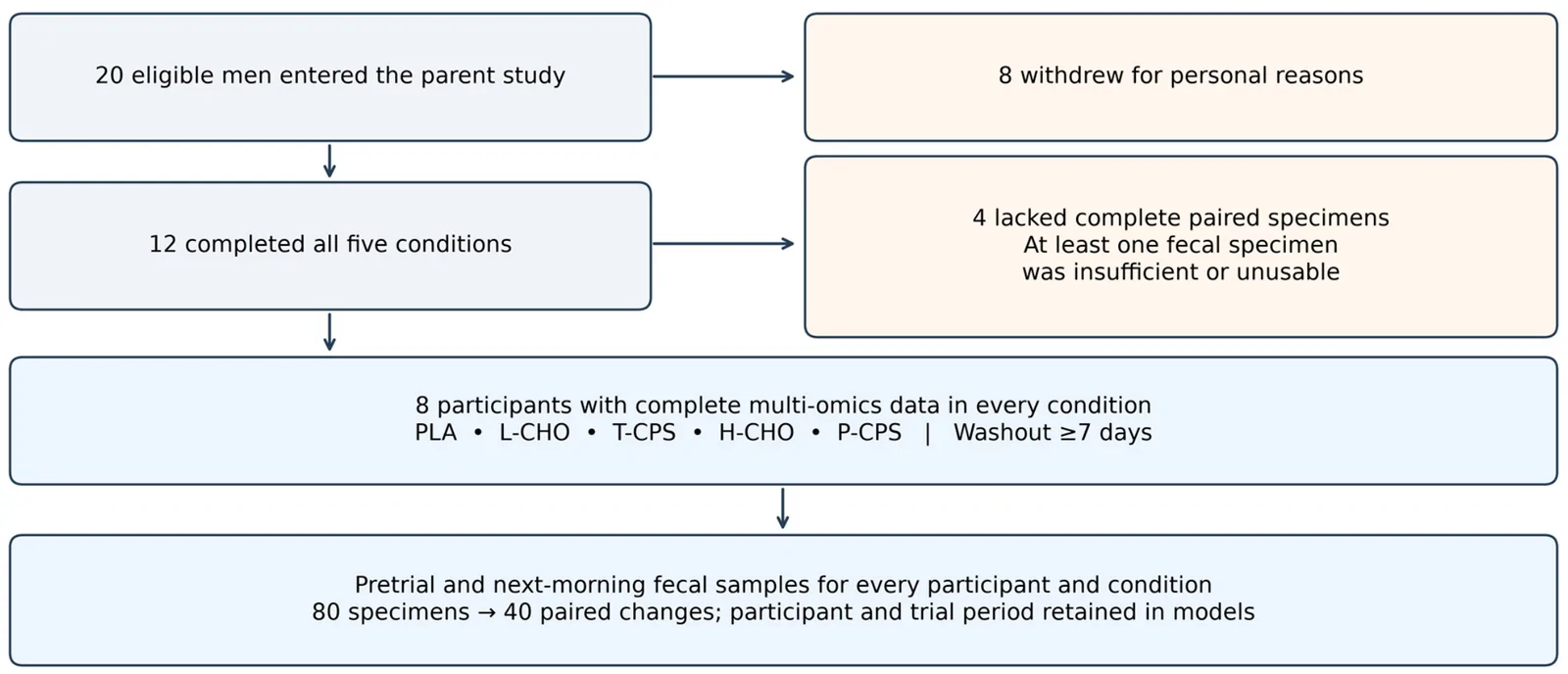
The study design and analysis flow. Twenty eligible men entered the parent study; eight withdrew for personal reasons and 12 completed five nutritional conditions. Four completers lacked complete paired data because at least one specimen was insufficient or unusable. The present complete-case multi-omics analysis included eight participants with paired pre-trial and next-morning fecal data under every condition. The resulting 80 specimens yielded 40 within-trial changes. Washouts were at least seven days. Downward arrows indicate participant progression and specimen collection, rightward arrows indicate withdrawals or exclusions, and the arrow from 80 specimens to 40 paired changes denotes pairing of pretrial and next-morning samples.

**Figure 2:**
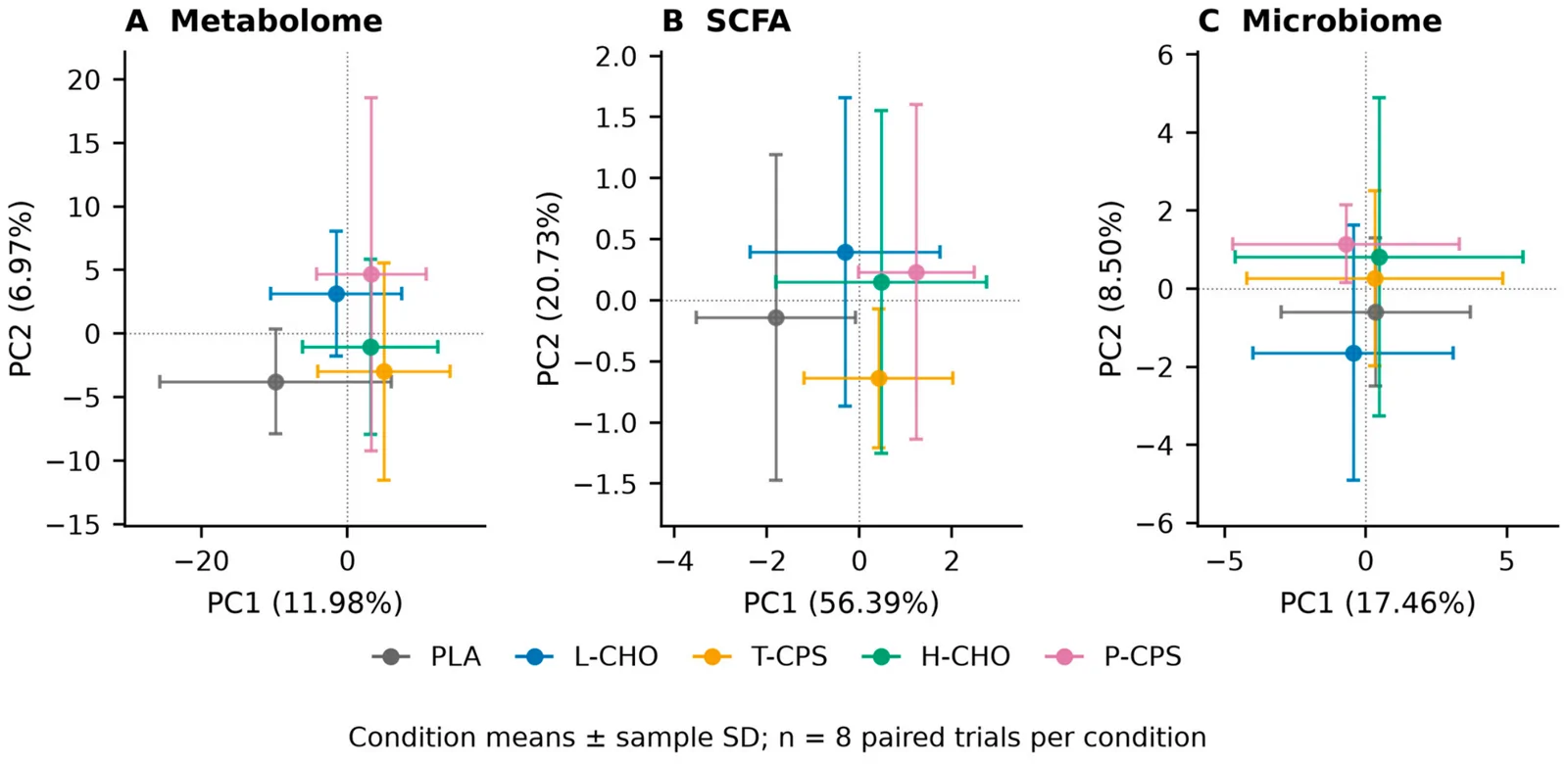
Descriptive PCA summaries of within-trial changes. The panels show (**A**) metabolomics, (**B**) SCFAs, and (**C**) the microbiome using the original provider annotations. The points represent condition means on the first two component axes; the horizontal and vertical bars show the sample SD of PC1 and PC2, respectively, across eight participants. The coordinates and explained variance derive from the original unsupervised PCA of 40 paired trials. The bars describe dispersion, not confidence intervals. These summaries do not replace the participant-blocked, period-adjusted global tests.

**Figure 3:**
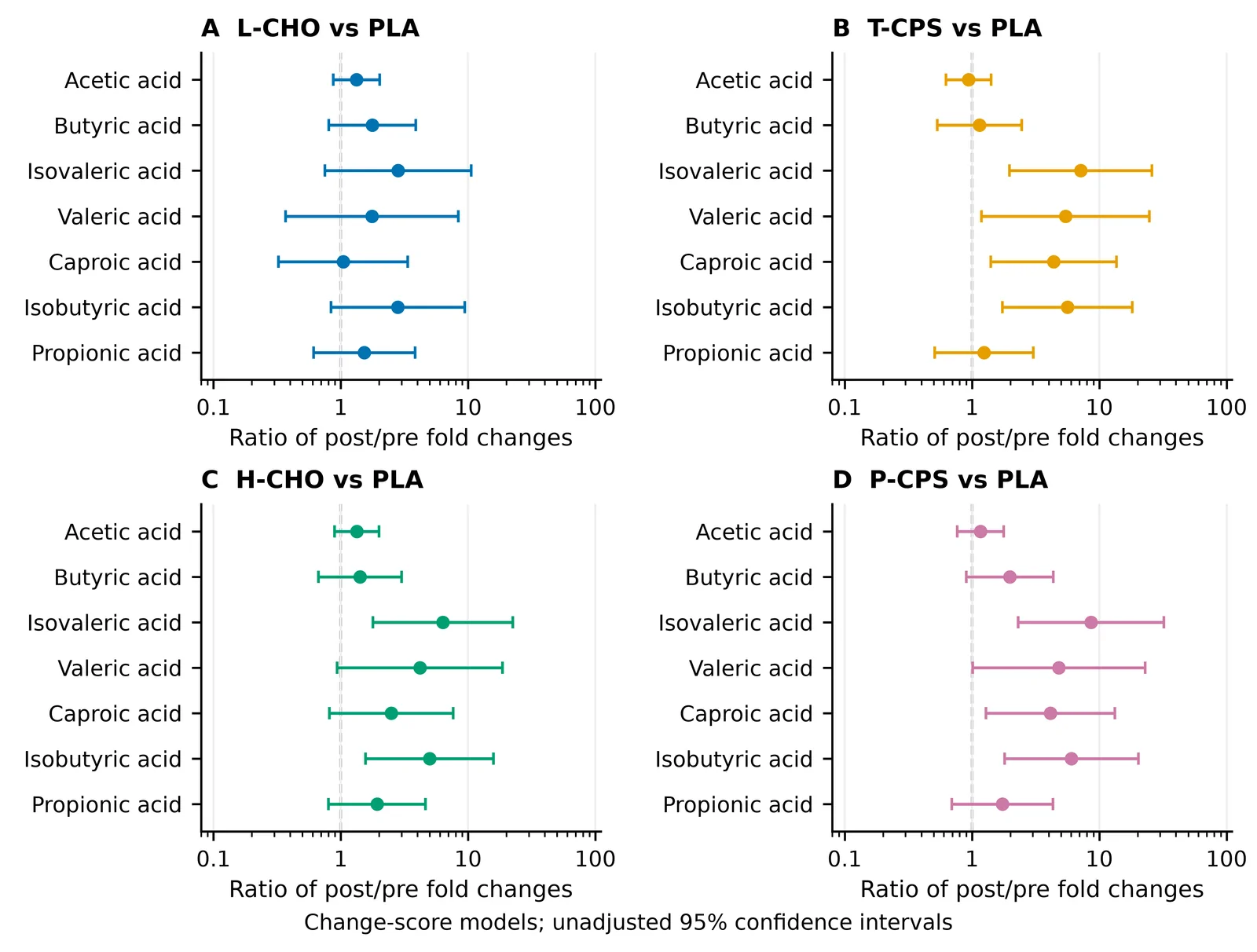
The complete active-condition-versus-PLA SCFA change-score contrasts. The panels compare (**A**) L-CHO, (**B**) T-CPS, (**C**) H-CHO, and (**D**) P-CPS with PLA. The points and bars show adjusted ratios of next-morning-to-pre-trial fold changes and unadjusted 95% confidence intervals from participant-blocked, period-adjusted models. The horizontal axes use a logarithmic scale; the dashed line indicates a ratio of one. Every comparison is displayed, including nonsignificant results. Complete *p* values and within-contrast and 28-comparison q values are provided in Table S9. Caproic acid includes observations at or below LOQ and requires particular caution. These secondary change-score findings were sensitive to the handling of pre-trial concentration: no concentration contrast remained FDR-positive in the baseline-adjusted models (Table S14).

**Figure 4:**
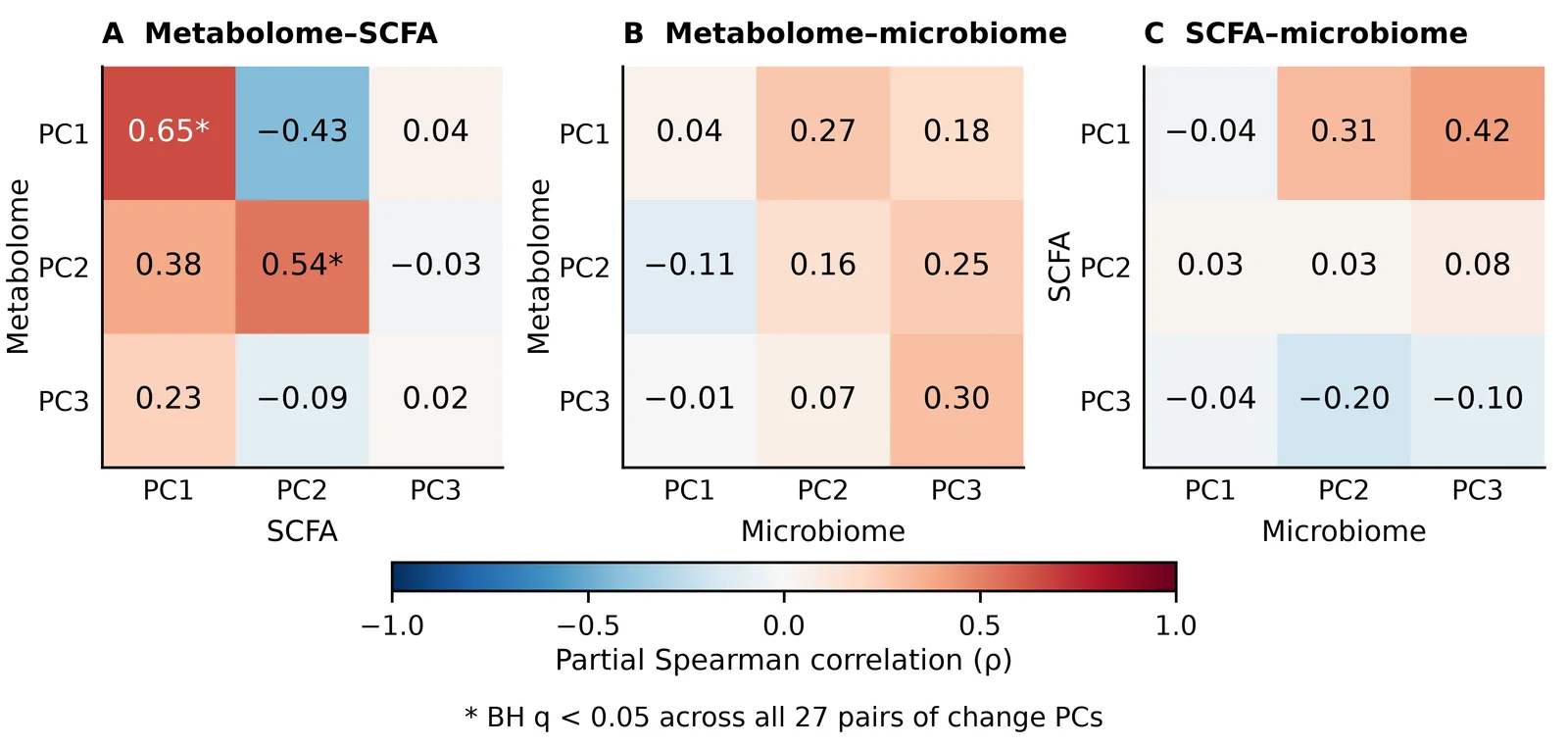
The cross-omics partial Spearman correlations of the paired changes. The matrices show all 27 pairs among the first three change PCs from the original annotation-based analyses: (**A**) metabolome–SCFA, (**B**) metabolome–microbiome, and (**C**) SCFA–microbiome. The values are partial correlation coefficients after rank transformation and adjustment for participant and categorical period. The asterisks identify BH q < 0.05 across all 27 tests using 4999 within-participant permutations. Intervention was not additionally residualized. Only metabolome–SCFA PC1–PC1 and PC2–PC2 passed this correction; these exploratory associations do not establish independent replication or a microbial mechanism.

**Table 1:** The characteristics of the eight-person multi-omics subset.

Characteristic	Mean (SD)	Range
Age, y	25.5 (2.1)	23–30
Height, cm	180.0 (3.9)	173–185
Body mass, kg	80.6 (8.6)	66.2–89.0
Body water, %	52.9 (1.8)	50.9–56.5
Body fat, %	23.5 (3.0)	17.6–26.6

Values are calculated for participants 1–8 in the parent crossover cohort and are not the full 12-person cohort summary.

**Table 2:** Primary global intervention tests with across-modality FDR and permutation-null partial R^2^.

Modality	Variables	Pseudo-F	*p*	q (3 Tests)	Partial R^2^	Null R^2^ Median/95th
Untargeted metabolome	1051	1.146	0.0889	0.1334	0.160	0.141/0.165
Targeted SCFAs	7	1.935	0.0321	0.0963	0.244	0.128/0.229
Genus CLR microbiome	88	1.067	0.2813	0.2813	0.151	0.141/0.170

The full models included participant, categorical period, and intervention; the reduced models omitted intervention. The tests used 9999 Freedman–Lane permutations restricted within participant (seed 20260806). All pseudo-F tests had 4 and 24 degrees of freedom. The q values adjust the three primary *p* values together. The null values are the median and 95th percentile of permutation partial R^2^. CLR, centered log ratio.

**Table 3:** Complete secondary change-score contrasts for P-CPS versus PLA across all seven fecal SCFAs.

Acid	Log_2_ Change Difference (95% CI)	Ratio of Fold Changes (95% CI)	*p*	q_7_	q_28_
Acetic acid	0.219 (−0.386 to 0.825)	1.16 (0.77–1.77)	0.4621	0.4621	0.5406
Propionic acid	0.794 (−0.527 to 2.116)	1.73 (0.69–4.34)	0.2268	0.2646	0.3175
Butyric acid	0.987 (−0.147 to 2.122)	1.98 (0.90–4.35)	0.0850	0.1190	0.1954
Isobutyric acid	2.596 (0.853 to 4.338)	6.04 (1.81–20.23)	0.0052	0.0182	0.0334
Isovaleric acid	3.107 (1.203 to 5.011)	8.62 (2.30–32.24)	0.0025	0.0178	0.0334
Valeric acid	2.266 (0.016 to 4.515)	4.81 (1.01–22.86)	0.0485	0.0849	0.1358
Caproic acid	2.046 (0.362 to 3.730)	4.13 (1.29–13.27)	0.0193	0.0451	0.0677

The estimates are participant-blocked, period-adjusted differences in log_2_ next-morning-to-pre-trial change. Exponentiation gives the ratio of post/pre fold changes; the confidence intervals are unadjusted 95% t intervals (24 residual degrees of freedom). q_7_ adjusts the seven original P-CPS-versus-PLA tests; q_28_ adjusts all four-active-condition-versus-PLA comparisons. Positive estimates indicate larger post/pre change under P-CPS. Caproic and valeric acid include values at or below LOQ; acetate includes one value above the listed upper calibration limit. No concentration contrast remained FDR-positive after same-trial pre-trial adjustment (Table S14).

## Data Availability

Participant-level raw and processed datasets are not publicly available because the confidentiality clause of the informed consent form signed by participants restricts access to their personal information and experimental data to the participant and research team. Unrestricted public deposition is not covered by that consent. The authors retain the sample-level 16S FASTQ files and converted targeted SCFA chromatograms; untargeted mass-spectrometry acquisition files were unavailable for the present reanalysis. No public raw-data accession number is available. Analysis code, input schemas, and aggregate statistical results are supplied in Supplementary Data S1. Questions regarding data access may be directed to the corresponding author. Any release of participant-level data would require authorization consistent with the signed consent; access cannot be guaranteed under the existing consent.

## Supplementary Materials

The following supporting information can be downloaded at https://www.mdpi.com/article/10.3390/metabo16100718/s1. Supporting information comprises S1: Supplementary Methods; Table S1: Number of participants in each condition by trial period; Table S2: Distribution of P-CPS prescription rates in the multi-omics subset; Table S3: Alpha-diversity intervention tests; Table S4: Complete P-CPS-versus-PLA SCFA contrasts; Table S5: Cross-omics PC correlations surviving FDR; Table S6: Beverage prescriptions and protocol-calculated cumulative intake; Table S7: Absolute fecal SCFA concentrations by condition and time point; Table S8: Extract-concentration quantification limits and analytical-range audit; Table S8a: Counts at or near LOQ by condition and time (n = 8 in each cell); Table S9: All four active-condition-versus-PLA SCFA contrasts (post hoc); Table S10: SCFA mass-composition and composite-endpoint analyses (post hoc); Table S11: Read counts, prevalence, and abundance for all 88 retained genus-level labels; Table S12: Two original-analysis chemical PC associations across annotation-exclusion scenarios; Table S13: Putative metabolomic annotations removed in the two sensitivity sets; Table S14: Same-trial baseline-adjusted SCFA sensitivity (post hoc); Table S15: Original P-CPS-versus-active-comparator SCFA contrasts on both scales; Table S16: Completed rowing distance by condition in the multi-omics subset and parent cohort; Table S17: SILVA 138.2 taxonomic sensitivity analyses (post hoc); Table S18: Retained untargeted-metabolomics annotation evidence; Figure S1: Absolute fecal SCFA concentrations summarized by condition and time; Figure S2: SCFA mass composition, total concentrations, and branched-acid percentage; CONSORT 2025 checklist with the 2019 crossover extension; Supplementary Data S1: Analysis Code and Summary Results. Participant-level input matrices and linked sample records are not included under the consent-based restrictions described in the Data Availability Statement. References [21,22,23,24,25,32,33,34,36,47] are cited in the supplementary materials.
