# Supplementary material for: Fecal Metabolic and Microbial Responses to Five Acute Fueling Strategies During a 60-Minute Rowing Protocol: A Randomized Crossover Multi-Omics Pilot Study
Source: Metabolites. 2026 Sep 28;16(10):718. doi: 10.3390/metabo16100718 (PMC13707109; doi:10.3390/metabo16100718)
Supplement: Supplementary file 1 [file metabolites-16-00718-s001.zip › metabolites-4529093_Rev2_CONSORT_Checklist.pdf]

CONSORT 2025 checklist of information to include when reporting a randomised trial*

Manuscript metabolites-4529093 — five-condition crossover study

Section / Topic	No	CONSORT 2025 checklist item description	Reported location and explanation
**Title and abstract**			
Title and structured abstract	1a	Identification as a randomised trial	Title.
	1b	Structured summary of the trial design, methods, results, and conclusions	Abstract. A structured summary is provided; not all crossover abstract items are available.
**Open science**			
Trial registration	2	Name of trial registry, identifying number (with URL) and date of registration	The parent trial was not registered. Section 2.1. Ethics approval 2022A57 is not trial registration.
Protocol and statistical analysis plan	3	Where the trial protocol and statistical analysis plan can be accessed	Sections 2.1–2.2 and 2.9; Supplementary Methods S1.2–S1.11; Supplementary Data S1. A prospective parent-trial protocol and analysis-plan access point are not reported.
Data sharing	4	Where and how the individual de-identified participant data (including data dictionary), statistical code and any other materials can be accessed	Data Availability Statement; Supplementary Methods S1.7; Supplementary Data S1. Code and aggregate results are supplied. Participant-level access is restricted by consent and cannot be guaranteed.
Funding and conflicts of interest	5a	Sources of funding and other support (e.g., supply of drugs), and role of funders in the design, conduct, analysis and reporting of the trial	Funding; Conflicts of Interest; Acknowledgments. Grant 25534510219; the funder had no role in the study or reporting.
	5b	Financial and other conflicts of interest of the manuscript authors	Conflicts of Interest.
**Introduction**			
Background and rationale	6	Scientific background and rationale	Section 1.

Section / Topic	No	CONSORT 2025 checklist item description	Reported location and explanation
Objectives	7	Specific objectives related to benefits and harms	Section 1; Section 2.9. Objectives concern fecal responses; no prospective harms objective is reported.
**Methods**			
Patient and public involvement	8	Details of patient or public involvement in the design, conduct and reporting of the trial	Patient or public involvement in design, conduct, or reporting is not documented in the retained records.
Trial design	9	Description of trial design including type of trial (e.g., parallel group, crossover), allocation ratio, and framework (e.g., superiority, equivalence, non-inferiority, exploratory)	Sections 2.1, 2.4, and 2.5; Figure 1; Table S1. Five conditions, one exposure each, with washouts of at least 7 days. Sequence-allocation restrictions are not documented.
Changes to trial protocol	10	Important changes to the trial after it commenced including any outcomes or analyses that were not prespecified, with reason	Sections 1, 2.2, 2.9, and 4; Supplementary Methods S1.3–S1.11. Secondary reanalysis and post hoc additions are identified. A complete parent-trial amendment history is not available.
Trial setting	11	Settings (e.g., community, hospital) and locations (e.g., countries, sites) where the trial was conducted	Section 2.3 describes the population. The data-collection setting and location are not explicitly reported.
Eligibility criteria	12a	Eligibility criteria for participants	Section 2.3.
	12b	If applicable, eligibility criteria for sites and for individuals delivering the interventions (e.g., surgeons, physiotherapists)	Site- or provider-specific eligibility criteria are not reported.
Intervention and comparator	13	Intervention and comparator with sufficient details to allow replication. If relevant, where additional materials describing the intervention and comparator (e.g., intervention manual) can be accessed	Section 2.4; Tables S2 and S6; references 6, 7, and 21. Nutrient rates, volumes, timing, and the personalization rule are reported; a full intervention manual is not supplied.

Section / Topic	No	CONSORT 2025 checklist item description	Reported location and explanation
Outcomes	14	Pre-specified primary and secondary outcomes, including the specific measurement variable (e.g., systolic blood pressure), analysis metric (e.g., change from baseline, final value, time to event), method of aggregation (e.g., median, proportion), and time point for each outcome	Sections 1 and 2.5–2.9; Supplementary Methods S1.2–S1.11. The original reanalysis plan and post hoc additions are distinguished; the parent trial was not registered.
Harms	15	How harms were defined and assessed (e.g., systematically, non-systematically)	Sections 2.4 and 4. Investigators reported no adverse reactions during administration. Systematic harms definitions and quantitative gastrointestinal scores are unavailable.
Sample size	16a	How sample size was determined, including all assumptions supporting the sample size calculation	Sections 2.1 and 4; Figure 1. No prospective calculation was performed for secondary omics outcomes; the eight-person set reflects complete specimen availability.
	16b	Explanation of any interim analyses and stopping guidelines	Interim analyses and stopping guidelines are not documented in the retained records.
Randomisation:			
Sequence generation	17a	Who generated the random allocation sequence and the method used	Section 2.1. Randomized order was recovered from the trial workbook; the sequence-generation method and generator are not documented.
	17b	Type of randomisation and details of any restriction (e.g., stratification, blocking and block size)	Section 2.1; Table S1. Randomization restrictions, blocking, and stratification are not documented; Table S1 describes realized counts.
Allocation concealment mechanism	18	Mechanism used to implement the random allocation sequence (e.g., central computer/telephone; sequentially numbered, opaque, sealed containers), describing any steps to conceal the sequence until interventions were assigned	The allocation-concealment mechanism before assignment is not documented in the retained records.

Section / Topic	No	CONSORT 2025 checklist item description	Reported location and explanation
Implementation	19	Whether the personnel who enrolled and those who assigned participants to the interventions had access to the random allocation sequence	Personnel responsible for enrollment and sequence assignment, and their access to the allocation sequence, are not documented.
Blinding	20a	Who was blinded after assignment to interventions (e.g., participants, care providers, outcome assessors, data analysts)	Condition labels were concealed; sensory matching and successful blinding could not be verified from the retained records. Sections 2.4 and 4.
	20b	If blinded, how blinding was achieved and description of the similarity of interventions	Condition labels were concealed; sensory matching and successful blinding could not be verified from the retained records. Sections 2.4 and 4.
Statistical methods	21a	Statistical methods used to compare groups for primary and secondary outcomes, including harms	Section 2.9; Supplementary Methods S1.2–S1.11. Within-participant changes, categorical period, restricted permutations, contrasts, and FDR families are specified.
	21b	Definition of who is included in each analysis (e.g., all randomised participants), and in which group	Sections 2.1, 2.5, and 3.1; Figure 1. Eight complete-case participants, 40 trial changes, 80 specimens; this is not an intention-to-treat analysis of all entrants.
	21c	How missing data were handled in the analysis	Sections 2.1 and 2.5–2.7; Section 3.1. Participants without full paired specimens were excluded; retained feature values required no imputation. Analytical-range flags are reported.
	21d	Methods for any additional analyses (e.g., subgroup and sensitivity analyses), distinguishing prespecified from post-hoc	Section 2.9; Supplementary Methods S1.3–S1.11. Original and post hoc sensitivities are distinguished, including baseline adjustment and updated taxonomy.

Section / Topic	No	CONSORT 2025 checklist item description	Reported location and explanation
**Results**			
Participant flow, including flow diagram	22a	For each group, the numbers of participants who were randomly assigned, received intended intervention, and were analysed for the primary outcome	Sections 2.1 and 3.1; Figure 1; Table S1. Twenty eligible entrants, 12 completers, eight analyzed. The number randomized and flow by assigned sequence are not established.
	22b	For each group, losses and exclusions after randomisation, together with reasons	Section 2.1; Figure 1. Eight personal withdrawals and four completers excluded for insufficient/unusable fecal specimens. Withdrawal timing relative to randomization is not documented.
Recruitment	23a	Dates defining the periods of recruitment and follow-up for outcomes of benefits and harms	Recruitment and outcome-follow-up calendar dates are not reported. Section 2.5 provides relative sampling times only.
	23b	If relevant, why the trial ended or was stopped	The reason for ending the trial, including whether it ended as planned, is not explicitly documented.
Intervention and comparator delivery	24a	Intervention and comparator as they were actually administered (e.g., where appropriate, who delivered the intervention/comparator, how participants adhered, whether they were delivered as intended [fidelity])	Section 2.4; Tables S2 and S6. Prescribed intake was consumed in six portions. Beverage-delivery personnel and the adherence-verification method are not reported.
	24b	Concomitant care received during the trial for each group	Section 2.4; Section 4. Usual diet/activity/sleep instructions are reported; quantitative logs, recovery meals, and recent antibiotic exposure are unavailable.
Baseline data	25	A table showing baseline demographic and clinical characteristics for each group	Section 2.3; Table 1; Tables S1 and S7. Overall subset characteristics and condition-specific pretrial SCFAs are reported; sequence/period baseline tables are not provided.

Section / Topic	No	CONSORT 2025 checklist item description	Reported location and explanation
Numbers analysed, outcomes and estimation	26	For each primary and secondary outcome, by group:<br>● the number of participants included in the analysis<br>● the number of participants with available data at the outcome time point<br>● result for each group, and the estimated effect size and its precision (such as 95% confidence interval)<br>● for binary outcomes, presentation of both absolute and relative effect size	Sections 3.1–3.6; Tables 2–3; Tables S3–S18; Supplementary Data S1. Eight participants per condition; within-person contrasts and 95% intervals are reported. No binary efficacy outcomes.
Harms	27	All harms or unintended events in each group	Sections 2.4 and 4. No adverse reactions were reported during administration; a systematic condition-by-period harms dataset is unavailable.
Ancillary analyses	28	Any other analyses performed, including subgroup and sensitivity analyses, distinguishing pre-specified from post-hoc	Sections 3.4–3.6; Tables S4 and S9–S18; Supplementary Data S1. Post hoc analyses include baseline and taxonomy sensitivities; all baseline-adjusted acid/composite contrasts are FDR-negative.
**Discussion**			
Interpretation	29	Interpretation consistent with results, balancing benefits and harms, and considering other relevant evidence	Sections 4 and 5.
Limitations	30	Trial limitations, addressing sources of potential bias, imprecision, generalisability, and, if relevant, multiplicity of analyses	Section 4. Selection, imprecision, multiplicity, model dependence, generalizability, and measurement limitations are discussed. Absence of carryover is not established.

*We strongly recommend reading this statement in conjunction with the CONSORT 2025 Explanation and Elaboration and/or the CONSORT 2025 Expanded Checklist for important clarifications on all the items. We also recommend reading relevant CONSORT extensions. See www.consort-spirit.org.

Citation: Hopewell S, Chan AW, Collins GS, Hróbjartsson A, Moher D, Schulz KF, et al. CONSORT 2025 Statement: updated guideline for reporting randomised trials. BMJ. 2025; 389:e081123. https://dx.doi.org/10.1136/bmj-2024-081123.

© 2025 Hopewell et al. This is an Open Access article distributed under the terms of the Creative Commons Attribution License (https://creativecommons.org/licenses/by/4.0/), which permits unrestricted use, distribution, and reproduction in any medium, provided the original work is properly cited.

CONSORT 2019 extension for randomized crossover trials

Section / Topic	No	CONSORT crossover checklist item description	Reported location and explanation
**Title and abstract**			
Title†	1a	Identification as a randomised crossover trial in the title	Title.
Abstract†	1b	Specify a crossover design and report all information outlined in table 2	Abstract. Crossover design, analyzed sample, methods, and findings are summarized. Several items in the original article's Table 2, including sequence-level randomization details, are not available.
**Introduction**			
Background‡	2a	Scientific background and explanation of rationale	Section 1.
Objectives‡	2b	Specific objectives or hypotheses	Section 1; Section 2.9. Objectives concern fecal responses; no prospective harms objective is reported.
**Methods**			
Trial design†	3a	Rationale for a crossover design. Description of the design features including allocation ratio, especially the number and duration of periods, duration of washout period, and consideration of carry over effect	Sections 1, 2.1, 2.4, and 2.5; Figure 1; Table S1. Five acute conditions with washouts of at least 7 days support within-person comparisons; washout rationale and absence of carryover are not established.
Change from protocol‡	3b	Important changes to methods after trial commencement (such as eligibility criteria), with reasons	Sections 1, 2.2, 2.9, and 4; Supplementary Methods S1.3–S1.11. Secondary reanalysis and post hoc additions are identified. A complete parent-trial amendment history is not available.
Participants‡	4a	Eligibility criteria for participants	Section 2.3.
Settings and location‡	4b	Settings and locations where the data were collected	Section 2.3 describes the population. The data-collection setting and location are not explicitly reported.

Section / Topic	No	CONSORT crossover checklist item description	Reported location and explanation
Interventions†	5	The interventions with sufficient details to allow replication, including how and when they were actually administered	Section 2.4; Tables S2 and S6; references 6, 7, and 21. Nutrient rates, volumes, timing, and the personalization rule are reported; a full intervention manual is not supplied. Protocol-specified intake was consumed.
Outcomes‡	6a	Completely defined prespecified primary and secondary outcome measures, including how and when they were assessed	Sections 1 and 2.5–2.9; Supplementary Methods S1.2–S1.11. The original reanalysis plan and post hoc additions are distinguished; the parent trial was not registered.
Changes to outcomes‡	6b	Any changes to trial outcomes after the trial commenced, with reasons	Sections 1, 2.2, 2.9, and 4; Supplementary Methods. Original reanalysis and post hoc additions are distinguished; a complete parent-trial outcome-amendment history is unavailable.
Sample size†	7a	How sample size was determined, accounting for within participant variability	Sections 2.1 and 4; Figure 1. No prospective calculation was performed for secondary omics outcomes; the eight-person set reflects complete specimen availability. No within-participant variance assumption was specified.
Interim analyses and stopping guidelines‡	7b	When applicable, explanation of any interim analyses and stopping guidelines	Interim analyses and stopping guidelines are not documented in the retained records.
**Randomization**			
Sequence generation‡	8a	Method used to generate the random allocation sequence	Section 2.1. Randomized order was recovered from the trial workbook; the sequence-generation method and generator are not documented.

Section / Topic	No	CONSORT crossover checklist item description	Reported location and explanation
Sequence generation‡	8b	Type of randomisation; details of any restriction (such as blocking and block size)	Section 2.1; Table S1. Randomization restrictions, blocking, and stratification are not documented; Table S1 describes realized counts.
Allocation concealment mechanism‡	9	Mechanism used to implement the random allocation sequence§ (such as sequentially numbered containers), describing any steps taken to conceal the sequence until interventions were assigned	The allocation-concealment mechanism before assignment is not documented in the retained records.
Implementation†	10	Who generated the random allocation sequence,§ who enrolled participants, and who assigned participants to the sequence of interventions	Section 2.1. The identities of the sequence generator, enrollment personnel, and sequence-assignment personnel are not documented.
Blinding‡	11a	If done, who was blinded after assignment to interventions (for example, participants, care providers, those assessing outcomes) and how	Condition labels were concealed; sensory matching and successful blinding could not be verified from the retained records. Sections 2.4 and 4.
Similarity of interventions‡	11b	If relevant, description of the similarity of interventions	Condition labels were concealed; sensory matching and successful blinding could not be verified from the retained records. Sections 2.4 and 4.
Statistical methods†	12a	Statistical methods used to compare groups for primary and secondary outcomes which are appropriate for crossover design (that is, based on within participant comparison)	Section 2.9; Supplementary Methods S1.2–S1.11. Within-participant changes, categorical period, restricted permutations, contrasts, and FDR families are specified.
Additional analyses‡	12b	Methods for additional analyses, such as subgroup analyses and adjusted analyses	Section 2.9; Supplementary Methods S1.3–S1.11. Original and post hoc sensitivities are distinguished, including baseline adjustment and updated taxonomy.

Section / Topic	No	CONSORT crossover checklist item description	Reported location and explanation
**Results**			
Participant flow (a diagram is strongly recommended)†	13a	The numbers of participants who were randomly assigned, received intended treatment, and were analysed for the primary outcome, separately for each sequence and period	Sections 2.1 and 3.1; Figure 1; Table S1. Twenty eligible entrants, 12 completers, eight analyzed. The number randomized and flow by assigned sequence are not established.
Losses and exclusions†	13b	No of participants excluded at each stage, with reasons, separately for each sequence and period	Section 2.1; Figure 1. Eight personal withdrawals and four completers excluded for insufficient/unusable fecal specimens. Withdrawal timing relative to randomization is not documented. Losses by sequence and period are unavailable.
Recruitment‡	14a	Dates defining the periods of recruitment and follow-up	Recruitment and outcome-follow-up calendar dates are not reported. Section 2.5 provides relative sampling times only.
Trial end‡	14b	Why the trial ended or was stopped	The reason for ending the trial, including whether it ended as planned, is not explicitly documented.
Baseline data†	15	A table showing baseline demographic and clinical characteristics by sequence and period	Section 2.3; Table 1; Tables S1 and S7. Overall subset characteristics and condition-specific pretrial SCFAs are reported; sequence/period baseline tables are not provided.
Numbers analysed†	16	Number of participants (denominator) included in each analysis and whether the analysis was by original assigned groups	Sections 2.1, 2.5, and 3.1; Figure 1. Eight complete-case participants, 40 trial changes, 80 specimens; this is not an intention-to-treat analysis of all entrants.

Section / Topic	No	CONSORT crossover checklist item description	Reported location and explanation
Outcomes and estimation†	17a	For each primary and secondary outcome, results including estimated effect size and its precision (such as 95% confidence interval) should be based on within participant comparisons.¶ In addition, results for each intervention in each period are recommended	Sections 3.2–3.6; Tables 2–3; Tables S4, S7, and S9–S18; Supplementary Data S1. Scalar within-person effects include 95% intervals. Intervention-by-period outcome summaries are not provided.
Binary outcomes‡	17b	For binary outcomes, presentation of both absolute and relative effect sizes is recommended	Not applicable. No binary efficacy endpoint is analyzed.
Ancillary analyses‡	18	Results of any other analyses performed, including subgroup analyses and adjusted analyses, distinguishing prespecified from exploratory	Sections 3.4–3.6; Tables S4 and S9–S18; Supplementary Data S1. Post hoc analyses include baseline and taxonomy sensitivities; all baseline-adjusted acid/composite contrasts are FDR-negative.
Harms†	19	Describe all important harms or untended effects in a way that accounts for the design (for specific guidance, see CONSORT for harms 32)	Sections 2.4 and 4. No adverse reactions were reported during administration; a systematic condition-by-period harms dataset is unavailable.
**Discussion**			
Limitations†	20	Trial limitations, addressing sources of potential bias, imprecision, and if relevant, multiplicity of analyses. Consider potential carry over effects	Section 4. Selection, imprecision, multiplicity, model dependence, generalizability, and measurement limitations are discussed. Absence of carryover is not established.
Generalisability‡	21	Generalisability (external validity, applicability) of the trial findings	Section 4. Applicability is limited by the small complete-case sample of male university recreational rowers.
Interpretation‡	22	Interpretation consistent with results, balancing benefits and harms, and considering other relevant evidence	Sections 4 and 5.

Section / Topic	No	CONSORT crossover checklist item description	Reported location and explanation
**Other information**			
Registration‡	23	Registration number and name of trial registry	The parent trial was not registered. Section 2.1. Ethics approval 2022A57 is not trial registration.
Protocol‡	24	Where the full trial protocol can be accessed, if available	Sections 2.1–2.2 and 2.9; Supplementary Methods S1.2–S1.11; Supplementary Data S1. A prospective parent-trial protocol and analysis-plan access point are not reported.
Funding‡	25	Sources of funding and other support (such as supply of drugs), role of funders	Funding; Conflicts of Interest; Acknowledgments. Grant 25534510219; the funder had no role in the study or reporting.

† Modified original CONSORT item. ‡ Unmodified CONSORT item. Locations are given as sections, figures, and tables; page numbers are optional in the original checklist.
§ Random sequence refers to a list of random orders, typically generated by computer. It is distinct from an individual participant's sequence of interventions.
¶ Within-participant comparisons account for correlation between repeated measurements because participants act as their own controls.
Table 2 in item 1b refers to the abstract checklist in the original Dwan et al. article. Reference 32 in item 19 is: Ioannidis JP, Evans SJ, Gøtzsche PC, et al. Better reporting of harms in randomized trials: an extension of the CONSORT statement. Ann Intern Med. 2004;141:781–788. doi:10.7326/0003-4819-141-10-200411160-00009.
Citation: Dwan K, Li T, Altman DG, Elbourne D. CONSORT 2010 statement: extension to randomised crossover trials. BMJ. 2019;366:l4378. doi:10.1136/bmj.l4378.
© 2019 Dwan et al. Reproduced under the Creative Commons Attribution Non Commercial 4.0 license (https://creativecommons.org/licenses/by-nc/4.0/).
