# Supplementary material for: Fecal Metabolic and Microbial Responses to Five Acute Fueling Strategies During a 60-Minute Rowing Protocol: A Randomized Crossover Multi-Omics Pilot Study
Source: Metabolites. 2026 Sep 28;16(10):718. doi: 10.3390/metabo16100718 (PMC13707109; doi:10.3390/metabo16100718)
Supplement: Supplementary file 1 [file metabolites-16-00718-s001.zip › metabolites-4529093_Rev2_Supplementary_Material_Clean.pdf]

Supplementary Materials

Article: Fecal Metabolic and Microbial Responses to Five Acute Fueling Strategies during a 60-Minute Rowing Protocol: A Randomized Crossover Multi-Omics Pilot Study

Authors: Xiangyu Wang and Jian Huang

S1. Supplementary Methods

S1.1. Sample-Code Interpretation

The original fecal identifiers encoded condition, time point, and participant. These identifiers were decoded once and joined to the recovered crossover sequence. The resulting key was then matched independently against the metabolite abundance columns, SCFA sample rows, full ASV table, rarefied ASV table, taxonomy-associated feature table, and FASTQ basenames. Presence was required on all three platforms before a paired change was calculated. The sample-to-trial mapping was retained with the restricted analysis records and is not included in the supplementary files; no sample label was reassigned on the basis of its outcome profile.

S1.2. Model Construction

For each feature, the full design matrix contained an intercept, seven participant indicators (participant 1 as reference), four trial-period indicators (period 1 as reference), and four intervention indicators (PLA as reference). The omnibus intervention test compared that matrix with a reduced matrix containing only participant and period. P-CPS contrasts were linear combinations of full-model coefficients. Residual degrees of freedom were 24. The same model structure was applied across modalities so that modality comparisons did not arise from different handling of the crossover design.

For each multivariate matrix, the observed statistic was

```
[(RSSreduced − RSSfull) / 4] / [RSSfull / 24],
```

where RSS was summed over all variables. Reduced-model residual vectors were permuted among the five periods within each participant and added back to reduced-model fitted values. The full and reduced models were then refitted. The permutation P value was `(b + 1)/(B + 1)`, where B=9,999 and b was the number of permuted statistics at least as large as observed. The random-number seed was fixed at 20260806.

S1.3. Sensitivity Analyses

The no-period model retained participant and intervention. Leave-one-participant-out analyses refitted the period-adjusted model eight times and counted the number of estimates sharing the sign of the full-sample estimate. Exact paired Wilcoxon tests compared the eight condition-specific changes for key SCFA contrasts. These sensitivity analyses were used to assess fragility and not as alternate routes to statistical significance.

S1.4. Primary Multiplicity and Null Partial R^2

Benjamini–Hochberg adjustment was applied jointly to the three primary global P values. The partial R^2 null distributions were retained from the same 9,999 restricted permutations, and their medians and 95th percentiles are reported with the observed values. Percentiles used linear interpolation. Metabolite and SCFA change variables were standardized; genus CLR variables were not additionally standardized for the trace test. The seed was 20260806. The three tests remained primary; additional analyses below were post hoc and do not constitute independent confirmation.

S1.5. Extraction Conditions, SCFA Quantification, and Composition

For both metabolomic and targeted SCFA extraction, the provider reported centrifugation at 12,000 rpm for 10 min at 4 °C using an H1850-R centrifuge. Rotor identity and effective radius were not specified, so relative centrifugal force could not be calculated from the available record.

Fecal contents were summarized as arithmetic mean and sample SD, median with first and third quartiles, and minimum–maximum, separately for each condition and time point (n=8). Units are µg/g of fecal sample as weighed, without dry-mass normalization. Both retained calibration tables gave LOQ=0.02 µg/mL for all seven extract concentrations. Near LOQ was defined as ≤2×LOQ, inclusive of values ≤LOQ. For six acids, fecal content was C×0.5×1000/m, where C is µg/mL and m is sample mass in mg. The provider formula for caproic acid included an additional factor of 1.5. All 560 conversions were reproduced within source-rounding tolerance. Conversion consistency does not validate measurements outside the calibration range. The calibration sets could not be assigned to individual samples; their LOQs agreed. Upper limits were 500 µg/mL, except caproic acid in the second set (250 µg/mL). Original positive estimates were retained with flags, without substitution.

All four active conditions were compared with PLA for all seven $\log_2$ SCFA changes. The model included participant and categorical trial period, with 24 residual degrees of freedom. Every contrast has an unadjusted 95% t interval, nominal P, BH q across its seven acids, and BH q across all 28 active-versus-PLA tests. Exponentiating a coefficient and its limits as 2^β produces a ratio of post/pre fold changes between conditions. It does not give a ratio of posttrial absolute concentrations. The seven acid-specific omnibus tests form a separate FDR family.

For mass composition, each measured concentration was divided by the sample sum and natural-log CLR transformed; no pseudocount was needed. The global CLR test used the unstandardized CLR change matrix and the same restricted permutation scheme. Seven CLR omnibus tests and 28 CLR contrasts formed separate FDR families. Composite endpoints were $\log_2$ total measured SCFA, the mass percentage of isobutyrate plus isovalerate in the seven-acid sum, and $\log_2$[(isobutyrate+isovalerate)/(acetate+propionate+butyrate)]. Their three omnibus tests and 12 active-versus-PLA contrasts formed separate FDR families. These are mass proportions, not molar proportions.

An additional analytical-range sensitivity omitted an entire acid if any observation was ≤LOQ. This removed caproic and valeric acid while retaining all 80 samples. The five-acid $\log_2$-change matrix was standardized for its global concentration test. A second test recomputed CLR across the five retained acids before forming changes. The two restricted-permutation P values were BH adjusted together. Both analyses remain supplementary to the original seven-acid model.

S1.6. Microbial Counts and Annotation-Exclusion Sensitivity

Genus aggregation used the unrarefied ASV table after chloroplast and mitochondrial removal. The provider's fallback labels, including unresolved higher-rank placeholders, were retained when genus assignment was unavailable. Each label required prevalence ≥20% and mean relative abundance ≥0.01%. Counts, including zeros, were summarized for all 88 retained labels. A post hoc test omitted Pseudomonas and Acinetobacter, added 0.5 to the remaining 86 counts, recomputed natural-log CLR, and repeated the original global test. This test evaluates numerical dependence, not contamination status.

Metabolomic data were $\log_2$ transformed and sample median centered separately by ionization mode. All 1,051 features had nonzero change variance; mode-qualified identifiers retained two distinct features sharing one vendor identifier. The precise exclusion set removed isovaleric- and caproic-acid annotations. The broad set additionally removed ten annotated nonaromatic C2–C6 free monocarboxylic acids containing only C, H, and O and ≤4 oxygen atoms. It included hydroxy, oxo, and semialdehyde analogues, but excluded dicarboxylic acids, amino acids, sugars/polyols, esters, lactones, and conjugates. Rules were fixed before computing these sensitivity results. Table S13 records each removed annotation.

Mode-specific centering, paired changes, and standardized PCA were repeated after each exclusion. Component order followed explained variance; only signs were aligned to the corresponding baseline component. All 27 correlations among the first three PCs of each modality pair were recalculated within each scenario. Rank scores were residualized for participant and period, with 4,999 within-participant permutations (seed 20260806). BH adjustment covered all 27 P values within each scenario, not only the two selected associations. It did not correct for selecting among sensitivity scenarios. Feature loadings, explained variance, axis-alignment coefficients, and all 81 correlation results are provided in Supplementary Data S1. Individual PC scores are not included.

S1.7. Analysis Code and Summary Results

Supplementary Data S1 contains analysis code, abstract input schemas, nonparticipant assay and feature annotations, aggregate summaries, and model-level results for the global multi-omics and SCFA analyses. It excludes individual-level inputs, sample-to-trial mappings, paired-change matrices, participant PC scores, and individual assay flags. The consent used in this study limited access to personal information and experimental data to each participant and the research team; third-party access is not offered under that consent. The scripts specify dependencies, transformations, models, and run commands but require independently authorized inputs for complete reproduction. Figures 2–4, S1, and S2 can be regenerated from the supplied aggregate tables without individual-level inputs. These materials do not replace acquisition data for upstream sequence or peak processing.

S1.8. Same-Trial Baseline Sensitivity and Complete SCFA Contrast Scales

Baseline sensitivity retained every participant, trial, and acid. For each acid, the outcome was $\log_2$ next-morning concentration and the predictors were an intercept, participant (seven indicators), period (four indicators), condition (four indicators, PLA reference), and the same-trial $\log_2$ pretrial concentration. The 40×17 design was full rank, leaving 23 residual degrees of freedom. Each acid had one common baseline slope across conditions. The three pre-existing composite endpoints used the same model structure on their original scales: $\log_2$ total SCFA, branched mass percentage, and $\log_2$ branched-to-major-straight-chain ratio. No endpoint was selected for this sensitivity on the basis of its adjusted result.

Two-sided 95% t intervals and P values were calculated from the fitted models. BH families covered all 28 acid contrasts, all 12 composite contrasts, seven acid omnibus tests, and three composite omnibus tests separately. For $\log_2$ outcomes, exponentiation yields a conditional next-morning ratio at a common pretrial value, not a ratio of post/pre fold changes. Modeling change with the same pretrial covariate gives identical intervention coefficients; only the baseline coefficient changes by one. The baseline covariate can also be centered within participant without changing these intervention contrasts. These checks and all model outputs are supplied with the code. The models are post hoc, assume a common endpoint-specific baseline slope, and do not replace the primary analysis.

Table S14 gives every acid and composite sensitivity contrast. Table S15 completes the fold-change-ratio presentation for the 21 original P-CPS-versus-active-comparator contrasts. Table S15 uses the original seven-acid contrast-specific q values, without redefining the multiplicity family or changing any P value. Together with Table S9, it covers all 49 distinct SCFA change-score contrasts reported in this revision.

S1.9. Contemporary Taxonomic Reclassification

The original taxonomy artifact was imported as a taxonomy table and did not retain the classifier run. The provider report identifies Greengenes release 13.8 as its default database, with SILVA 132 as an alternative. Bracketed family names and placeholder genera in the delivered labels are consistent with Greengenes 13_8, making it the probable original reference rather than a verified classifier provenance.

The first 100 reads from each of 80 forward and 80 reverse FASTQ files were inspected for read-start motifs. Of 8,000 forward reads, 7,733 exactly matched ACTCCTACGGGAGGCAGCAG and 7,945 matched with at most one mismatch. Of 8,000 reverse reads, 3,534 exactly matched the degenerate

motif GGACTACHVGGGTWTCTAAT, 7,634 matched with at most one mismatch, and 7,827 with at most two. IUPAC-degenerate positions were treated as allowed bases. These patterns support the 338F/806R V3–V4 pair, but do not replace the missing laboratory primer record. The retained representative ASVs had a median length of 406 bases.

All 35,735 representative ASVs were classified with the official SILVA 138.2 SSU Ref NR99 full-length, uniform-prior QIIME 2 release 2025.7 classifier (DOI: 10.82364/138.2/SSU/Ref-NR99/QIIME2/2025.7/full-length/uniform; artifact MD5: 5cadf251ad7112e4ef4e36f1e203b046). Its scikit-learn 1.4.2 model and q2-feature-classifier prediction and hierarchical confidence rules were used with confidence 0.7, automatic orientation, and batches of 500 ASVs. A Python adapter provided input and output handling; it did not retrain the model or change the prediction rules. This was a taxonomic reclassification of retained ASVs, not re-denoising of FASTQ reads.

Taxonomy was joined by ASV identifier to the original unrarefied 35,735-by-80 count table. Chloroplast and mitochondrial assignments were excluded. Aggregation used the full classified lineage through genus; assignments ending above genus retained their deepest available rank. Identically named unresolved categories from different lineages were not merged. Of 507 labels, 130 passed prevalence ≥20% and mean relative abundance ≥0.01%, including 24 unresolved categories. Counts received a 0.5 pseudocount before natural-log CLR transformation and next-morning minus pretrial differencing. Reference, classifier, and aggregation key changed together; this is not a database-only comparison.

The participant-blocked, categorical-period-adjusted design, residual degrees of freedom (24), 9,999 restricted Freedman–Lane permutations, and seed 20260806 were retained. BH correction covered the 130 omnibus tests and each of four separate 130-test P-CPS contrast families. The alternative global family substituted the SILVA global P value for the original microbiome P value while retaining the other two modality P values. It is reported separately and does not replace the original primary family. Original-annotation counts, CLR changes, the global P value, and all 352 original contrasts were reproduced before comparing the new results. Complete aggregate filtering, omnibus, and contrast results accompany the code in Supplementary Data S1.

S1.10. Metabolomic Annotation Evidence

The delivered annotation workbook contained 1,051 features, 17 annotation fields, and 80 abundance columns. All entries carried the provider code “level 2”, without a feature-specific MS2 acquisition flag or numerical spectral-match score. Every feature name mapped to an identification plot. Six inspected plots spanning all five library labels displayed experimental/reference mirror spectra; this limited inspection does not establish acquired-MS2 coverage across the matrix. The 137 MetaDNA entries were not assumed to be direct verified library matches. No new acquisition data or authentic-standard results were obtained.

The mass-error audit used the absolute value of the delivered annotation ppm field. It describes annotation matching, not instrument calibration performance: 175 entries exceeded 15 ppm, 397 exceeded 5 ppm, and the maximum was 29.9177 ppm. Accordingly, all compound names remain provisional candidate labels. MSI level 3 is appropriate only where compound-class evidence is available; otherwise the feature remains unidentified at MSI level 4. The original MSI standard and instrument specification are cited in manuscript Section 2.6. Table S18 summarizes the evidence without treating plot availability or the provider code as verified MSI level 2.

S1.11. Descriptive Exercise Exposure

Completed rowing distance was extracted from the contemporaneous trial worksheet for all 60 trials in the 12-person parent cohort. The 40 entries for the eight-person multi-omics subset matched its trial index by participant and condition. Distance is the total across the two 30-min rowing bouts; the 15-min seated rest contributes no rowing distance. Arithmetic means and sample standard deviations (n−1 denominator) were calculated separately for the eight-person subset and 12-person cohort. Parent-cohort values agree with the doctoral dissertation after rounding. Table S16 presents descriptive exposure information without additional performance hypothesis tests.

Table S1. Number of participants in each condition by trial period.

Period	PLA	L-CHO	T-CPS	H-CHO	P-CPS	Total
1	2	1	1	1	3	8
2	1	1	2	1	3	8
3	3	1	2	2	0	8
4	1	4	0	2	1	8
5	1	1	3	2	1	8

Entries are counts of participants receiving each condition in each period. Each period contains eight participants, and each condition was received by all eight participants across the five periods. Participant-specific sequences and sample mappings are not included.

Table S2. Distribution of P-CPS prescription rates in the multi-omics subset.

Prescription	Unit	n	Mean ± SD	Minimum–maximum
Carbohydrate	$g \cdot kg^{-1} \cdot h^{-1}$	8	0.6125 ± 0.0968	0.50–0.77
Protein	$g \cdot kg^{-1} \cdot h^{-1}$	8	0.1538 ± 0.0226	0.13–0.19
Estimated energy	$kcal \cdot kg^{-1} \cdot h^{-1}$	8	3.0650 ± 0.4771	2.52–3.84

These summaries describe the prescription rates administered to the eight participants in the multi-omics subset. Estimated energy uses 4 kcal/g for carbohydrate and protein. Table S6 reports cumulative quantities calculated over the 75-min protocol. Participant-specific prescriptions are not included.

Table S3. Alpha-diversity intervention tests

Metric	Raw *P*	FDR *q*	Partial η^2
Observed features	0.884	0.884	0.045
Shannon	0.599	0.834	0.105
Simpson	0.275	0.687	0.186
Chao1	0.668	0.834	0.091
Good's coverage	0.163	0.687	0.230

Table S4. Complete P-CPS-versus-PLA SCFA contrasts

Analyte	Adjusted $\log_2$ difference (95% CI)	Period-adjusted q_7	No-period *q*	Exact Wilcoxon FDR *q*	LOO sign
Isovaleric acid	3.107 (1.203 to 5.011)	0.0178	0.0056	0.0273	8/8
Isobutyric acid	2.596 (0.853 to 4.338)	0.0182	0.0056	0.0273	8/8
Caproic acid	2.046 (0.362 to 3.730)	0.0451	0.0179	0.0547	8/8
Valeric acid	2.266 (0.016 to 4.515)	0.0849	0.0203	0.0365	8/8
Acetic acid	0.219 (−0.386 to 0.825)	0.4621	0.1573	0.3828	8/8
Butyric acid	0.987 (−0.147 to 2.122)	0.1190	0.0426	0.0547	8/8
Propionic acid	0.794 (−0.527 to 2.116)	0.2646	0.1218	0.2279	8/8

LOO sign is the number of eight leave-one-participant-out estimates with the same direction as the full-sample estimate. Divergence between modeled, no-period, and rank-based results is displayed rather than resolved by selecting the smallest P value. Period-adjusted, no-period, and exact Wilcoxon q

values each adjust the seven P-CPS-versus-PLA tests within their respective analysis. The expanded q across all 28 active-versus-PLA comparisons is reported separately in Table S9.

Table S5. Cross-omics PC correlations surviving FDR

Variables	Partial Spearman ϱ	Restricted *P*	FDR *q*	LOO sign
Metabolome PC1–SCFA PC1	0.653	0.0002	0.0054	8/8
Metabolome PC2–SCFA PC2	0.538	0.0014	0.0189	8/8

Complete model-level results for all tested features, PC correlations, and sensitivity analyses are provided in Supplementary Data S1. Individual-level input matrices and mappings are not included. The 82 previously selected feature-level correlations are not used for inference; the complete unselected modality-level PC analyses support the reported cross-omics results.

Table S6. Beverage prescriptions and protocol-calculated cumulative intake.

(A) Prescription rates and beverage characteristics.

Cond.	CHO rate	Protein rate	Estimated energy rate	Volume	Osmolality
PLA	No CHO supplement	No protein supplement	Not calculated	900 mL	Not measured
L-CHO	0.80	0	3.20	900 mL	Not measured
T-CPS	0.80	0.20	4.00	900 mL	Not measured
H-CHO	1.00	0	4.00	900 mL	Not measured
P-CPS	0.50–0.77 (mean 0.6125)	0.13–0.19 (mean 0.1538)	2.52–3.84 (mean 3.065)	900 mL	Not measured

CHO and protein rates are $g \cdot kg^{-1} \cdot h^{-1}$; energy rates for active beverages are $kcal \cdot kg^{-1} \cdot h^{-1}$, calculated at 4 kcal/g for carbohydrate and protein. Table S2 summarizes the distribution of P-CPS rates.

(B) Cumulative nutrient and energy quantities per kilogram of body mass over 75 min.

Condition	Total CHO (g/kg)	Total protein (g/kg)	CHO/protein-derived energy (kcal/kg)
PLA	0	0	0 from CHO/protein
L-CHO	1.000	0	4.00
T-CPS	1.000	0.250	5.00
H-CHO	1.250	0	5.00
P-CPS	0.625–0.963 (mean 0.766)	0.163–0.238 (mean 0.192)	3.15–4.80 (mean 3.83)

Cumulative quantities are calculated from the reported, rounded prescriptions over the 75-min protocol: rate × 75/60 h. The original carbohydrate and protein values were retained separately. The 75 min comprised two 30-min rowing bouts separated by 15 min of seated rest. Multiplying a cumulative g/kg value by body mass in kilograms gives total grams; dividing by six gives grams per 150-mL portion. P-CPS summaries were calculated from individual prescriptions before aggregation. All beverages were consumed in full as six 150-mL portions at 0, 15, 30, 45, 60, and 75 min. PLA contained 4.5 g erythritol in 900 mL (0.5% w/v), or 0.75 g per portion. Energy covers carbohydrate and protein; no value is assigned to erythritol. Osmolality was not measured for any beverage.

Table S7. Absolute fecal SCFA concentrations by condition and time point.

Each row summarizes eight participants. Values are µg/g of fecal sample as weighed. Q1 and Q3 are the first and third quartiles. Original positive estimates are retained, including values with analytical-range flags listed in Table S8. Figure S1 displays group medians and interquartile ranges; the corresponding aggregate summaries are supplied in Supplementary Data S1.

Acetic acid

Cond.	Time	Mean ± SD	Median (Q1–Q3)	Range
PLA	Pretrial	2768.56 ± 993.88	2725.53 (1892.37–3618.46)	1461.01–4194.53
PLA	Next morning	2113.44 ± 670.22	2077.63 (1604.66–2300.88)	1466.52–3533.24
L-CHO	Pretrial	2393.37 ± 684.34	2435.22 (2153.57–2680.16)	1370.16–3412.53
L-CHO	Next morning	2504.09 ± 722.23	2455.46 (2118.37–3151.08)	1255.98–3295.84
T-CPS	Pretrial	3021.28 ± 517.84	2911.23 (2679.89–3362.28)	2419.98–3836.84
T-CPS	Next morning	2528.26 ± 572.59	2478.75 (2064.74–2789.94)	1921.32–3392.25
H-CHO	Pretrial	2425.50 ± 467.60	2499.98 (2349.37–2594.57)	1445.41–3105.81
H-CHO	Next morning	2776.87 ± 1224.35	2737.30 (1731.56–3379.05)	1331.91–5060.54
P-CPS	Pretrial	2384.40 ± 987.40	2243.09 (1745.70–2673.78)	1331.94–4381.45
P-CPS	Next morning	2450.82 ± 681.64	2432.42 (2052.62–2969.39)	1439.98–3370.82

Propionic acid

Cond.	Time	Mean ± SD	Median (Q1–Q3)	Range
PLA	Pretrial	1025.36 ± 315.46	1017.31 (732.48–1227.37)	674.10–1539.74
PLA	Next morning	773.29 ± 416.03	812.82 (557.32–955.07)	27.92–1439.21
L-CHO	Pretrial	1302.67 ± 690.83	1193.89 (1087.62–1307.03)	429.26–2857.62
L-CHO	Next morning	1038.57 ± 332.21	1112.28 (758.52–1228.64)	599.79–1564.94
T-CPS	Pretrial	1268.89 ± 490.62	1083.11 (903.58–1581.99)	778.14–1997.86
T-CPS	Next morning	950.69 ± 360.71	1137.35 (731.98–1198.58)	372.77–1258.72
H-CHO	Pretrial	1004.84 ± 341.55	1008.36 (725.67–1256.99)	586.81–1527.22
H-CHO	Next morning	1129.48 ± 422.45	1199.08 (1012.57–1350.95)	338.29–1717.97
P-CPS	Pretrial	1037.79 ± 549.01	896.57 (590.74–1546.65)	397.62–1735.81
P-CPS	Next morning	987.76 ± 140.84	963.75 (876.30–1117.01)	806.49–1179.61

Butyric acid

Cond.	Time	Mean ± SD	Median (Q1–Q3)	Range
PLA	Pretrial	1149.27 ± 383.49	1168.90 (904.93–1457.65)	578.14–1630.41
PLA	Next morning	775.96 ± 390.14	808.42 (578.05–983.13)	203.17–1350.45
L-CHO	Pretrial	790.93 ± 306.22	675.51 (568.24–1126.39)	425.12–1167.29
L-CHO	Next morning	858.44 ± 393.88	919.11 (649.99–1039.59)	189.31–1497.80
T-CPS	Pretrial	1006.28 ± 393.44	1037.63 (681.30–1274.66)	446.21–1630.71
T-CPS	Next morning	694.82 ± 232.07	642.61 (613.28–801.19)	324.26–1132.86
H-CHO	Pretrial	1173.59 ± 396.60	1316.99 (975.11–1463.07)	467.95–1544.71
H-CHO	Next morning	1170.76 ± 866.27	904.63 (683.89–1480.15)	189.05–3008.21
P-CPS	Pretrial	794.07 ± 363.20	763.06 (596.37–914.89)	261.87–1397.11
P-CPS	Next morning	979.09 ± 239.38	917.94 (834.83–1183.50)	638.51–1321.45

Isobutyric acid

Cond.	Time	Mean ± SD	Median (Q1–Q3)	Range
PLA	Pretrial	87.91 ± 71.70	63.27 (44.02–104.28)	20.66–226.12
PLA	Next morning	44.29 ± 31.54	50.52 (13.61–62.88)	2.76–88.13
L-CHO	Pretrial	71.84 ± 46.24	74.34 (35.44–111.10)	2.23–133.06
L-CHO	Next morning	76.81 ± 41.79	67.74 (42.69–95.43)	36.52–153.97
T-CPS	Pretrial	29.15 ± 11.63	26.39 (22.64–36.95)	13.41–50.03
T-CPS	Next morning	93.62 ± 85.95	56.07 (35.97–134.38)	15.92–267.16
H-CHO	Pretrial	55.85 ± 36.69	73.05 (26.22–78.13)	1.42–99.71
H-CHO	Next morning	91.16 ± 59.23	72.03 (55.46–120.50)	26.20–191.99
P-CPS	Pretrial	33.93 ± 18.78	42.32 (21.95–44.37)	2.60–56.17
P-CPS	Next morning	93.29 ± 48.57	77.06 (66.54–121.23)	36.90–181.01

Isovaleric acid

Cond.	Time	Mean ± SD	Median (Q1–Q3)	Range
PLA	Pretrial	89.29 ± 85.00	57.84 (31.94–108.82)	17.63–248.46
PLA	Next morning	35.78 ± 26.79	33.65 (11.15–56.67)	4.52–75.00
L-CHO	Pretrial	70.56 ± 53.90	59.64 (28.61–114.41)	1.39–155.55
L-CHO	Next morning	63.80 ± 37.71	52.54 (33.00–82.16)	30.63–122.94
T-CPS	Pretrial	22.27 ± 10.01	20.47 (18.43–23.67)	8.70–43.86
T-CPS	Next morning	81.93 ± 77.68	52.96 (29.65–119.11)	11.99–243.17
H-CHO	Pretrial	51.08 ± 34.59	71.31 (21.34–74.59)	0.76–83.64
H-CHO	Next morning	87.22 ± 64.06	61.01 (44.57–117.14)	24.82–198.96
P-CPS	Pretrial	26.97 ± 17.91	29.59 (13.22–41.66)	1.80–50.23
P-CPS	Next morning	84.24 ± 48.54	74.86 (46.58–120.87)	28.03–163.86

Valeric acid

Cond.	Time	Mean ± SD	Median (Q1–Q3)	Range
PLA	Pretrial	156.27 ± 130.40	140.45 (85.98–208.11)	7.76–418.17
PLA	Next morning	81.45 ± 61.20	85.06 (37.65–128.67)	0.17–157.09
L-CHO	Pretrial	182.16 ± 114.12	191.38 (116.08–263.88)	3.79–342.03
L-CHO	Next morning	103.89 ± 46.91	101.77 (81.79–138.08)	18.82–166.95
T-CPS	Pretrial	58.27 ± 48.50	51.24 (16.02–80.64)	11.63–146.58
T-CPS	Next morning	109.98 ± 83.65	95.14 (43.92–160.32)	23.93–241.51
H-CHO	Pretrial	93.26 ± 80.49	72.56 (20.80–168.19)	8.99–201.93
H-CHO	Next morning	129.78 ± 100.05	134.59 (31.94–184.24)	26.75–309.08
P-CPS	Pretrial	55.25 ± 44.28	51.93 (23.40–68.52)	5.28–141.27
P-CPS	Next morning	125.57 ± 82.58	118.74 (71.14–167.46)	18.56–242.08

Caproic acid

Cond.	Time	Mean ± SD	Median (Q1–Q3)	Range
PLA	Pretrial	9.61 ± 18.60	2.61 (0.57–6.32)	0.36–54.88
PLA	Next morning	6.45 ± 12.92	0.61 (0.35–3.96)	0.05–37.15
L-CHO	Pretrial	5.26 ± 8.98	1.29 (0.59–4.92)	0.33–26.66
L-CHO	Next morning	1.56 ± 1.86	0.83 (0.53–1.46)	0.36–5.89
T-CPS	Pretrial	0.74 ± 0.65	0.47 (0.27–1.04)	0.19–1.76
T-CPS	Next morning	1.11 ± 0.61	0.96 (0.62–1.42)	0.53–2.07
H-CHO	Pretrial	2.82 ± 6.00	0.74 (0.47–1.02)	0.21–17.65
H-CHO	Next morning	1.56 ± 1.52	1.46 (0.48–1.60)	0.29–5.02
P-CPS	Pretrial	1.03 ± 1.34	0.56 (0.32–0.87)	0.21–4.19
P-CPS	Next morning	1.45 ± 1.23	0.97 (0.77–1.58)	0.56–4.23

Table S8. Extract-concentration quantification limits and analytical-range audit.

All seven documented LOQs were 0.02 µg/mL. Near LOQ means ≤0.04 µg/mL and includes observations ≤LOQ. Fractions below use all 80 observations for each acid. Upper limits were 500 µg/mL, except caproic acid in one calibration set (250 µg/mL). A sample-to-calibration-set mapping was unavailable; the common lower threshold is unchanged.

Acid	Extract range (µg/mL)	≤LOQ, n/80 (%)	≤2×LOQ, n/80 (%)	Above upper limit
Acetic acid	125.5977–519.2115	0/80 (0%)	0/80 (0%)	1
Butyric acid	18.6027–308.6427	0/80 (0%)	0/80 (0%)	0
Caproic acid	0.0033–3.7024	8/80 (10%)	31/80 (38.75%)	0
Isobutyric acid	0.1395–27.4104	0/80 (0%)	0/80 (0%)	0
Isovaleric acid	0.0747–25.3426	0/80 (0%)	0/80 (0%)	0
Propionic acid	2.8257–289.1914	0/80 (0%)	0/80 (0%)	0
Valeric acid	0.0172–42.6537	1/80 (1.25%)	1/80 (1.25%)	0

One H-CHO next-morning acetate value was 519.2115 µg/mL, corresponding to 5,060.5414 µg/g. Sample-specific dilution or repeat-analysis documentation was not recovered. No caproic acid value exceeded 250 µg/mL. Table S8a provides lower-range counts by condition and time. Aggregate analytical-range summaries are supplied in Supplementary Data S1; individual assay flags and mass conversions remain in the restricted records.

Table S8a. Counts at or near LOQ by condition and time (n=8 in each cell).

Acid	Condition	Pre ≤LOQ	Post ≤LOQ	Pre ≤2×LOQ	Post ≤2×LOQ
Acetic acid	PLA	0/8 (0%)	0/8 (0%)	0/8 (0%)	0/8 (0%)
Acetic acid	L-CHO	0/8 (0%)	0/8 (0%)	0/8 (0%)	0/8 (0%)
Acetic acid	T-CPS	0/8 (0%)	0/8 (0%)	0/8 (0%)	0/8 (0%)
Acetic acid	H-CHO	0/8 (0%)	0/8 (0%)	0/8 (0%)	0/8 (0%)
Acetic acid	P-CPS	0/8 (0%)	0/8 (0%)	0/8 (0%)	0/8 (0%)
Propionic acid	PLA	0/8 (0%)	0/8 (0%)	0/8 (0%)	0/8 (0%)
Propionic acid	L-CHO	0/8 (0%)	0/8 (0%)	0/8 (0%)	0/8 (0%)
Propionic acid	T-CPS	0/8 (0%)	0/8 (0%)	0/8 (0%)	0/8 (0%)
Propionic acid	H-CHO	0/8 (0%)	0/8 (0%)	0/8 (0%)	0/8 (0%)
Propionic acid	P-CPS	0/8 (0%)	0/8 (0%)	0/8 (0%)	0/8 (0%)
Butyric acid	PLA	0/8 (0%)	0/8 (0%)	0/8 (0%)	0/8 (0%)
Butyric acid	L-CHO	0/8 (0%)	0/8 (0%)	0/8 (0%)	0/8 (0%)
Butyric acid	T-CPS	0/8 (0%)	0/8 (0%)	0/8 (0%)	0/8 (0%)
Butyric acid	H-CHO	0/8 (0%)	0/8 (0%)	0/8 (0%)	0/8 (0%)
Butyric acid	P-CPS	0/8 (0%)	0/8 (0%)	0/8 (0%)	0/8 (0%)
Isobutyric acid	PLA	0/8 (0%)	0/8 (0%)	0/8 (0%)	0/8 (0%)
Isobutyric acid	L-CHO	0/8 (0%)	0/8 (0%)	0/8 (0%)	0/8 (0%)
Isobutyric acid	T-CPS	0/8 (0%)	0/8 (0%)	0/8 (0%)	0/8 (0%)
Isobutyric acid	H-CHO	0/8 (0%)	0/8 (0%)	0/8 (0%)	0/8 (0%)
Isobutyric acid	P-CPS	0/8 (0%)	0/8 (0%)	0/8 (0%)	0/8 (0%)
Isovaleric acid	PLA	0/8 (0%)	0/8 (0%)	0/8 (0%)	0/8 (0%)
Isovaleric acid	L-CHO	0/8 (0%)	0/8 (0%)	0/8 (0%)	0/8 (0%)
Isovaleric acid	T-CPS	0/8 (0%)	0/8 (0%)	0/8 (0%)	0/8 (0%)
Isovaleric acid	H-CHO	0/8 (0%)	0/8 (0%)	0/8 (0%)	0/8 (0%)
Isovaleric acid	P-CPS	0/8 (0%)	0/8 (0%)	0/8 (0%)	0/8 (0%)
Valeric acid	PLA	0/8 (0%)	1/8 (12.5%)	0/8 (0%)	1/8 (12.5%)
Valeric acid	L-CHO	0/8 (0%)	0/8 (0%)	0/8 (0%)	0/8 (0%)

Acid	Condition	Pre ≤LOQ	Post ≤LOQ	Pre ≤2×LOQ	Post ≤2×LOQ
Valeric acid	T-CPS	0/8 (0%)	0/8 (0%)	0/8 (0%)	0/8 (0%)
Valeric acid	H-CHO	0/8 (0%)	0/8 (0%)	0/8 (0%)	0/8 (0%)
Valeric acid	P-CPS	0/8 (0%)	0/8 (0%)	0/8 (0%)	0/8 (0%)
Caproic acid	PLA	0/8 (0%)	1/8 (12.5%)	3/8 (37.5%)	4/8 (50%)
Caproic acid	L-CHO	0/8 (0%)	0/8 (0%)	2/8 (25%)	3/8 (37.5%)
Caproic acid	T-CPS	3/8 (37.5%)	0/8 (0%)	5/8 (62.5%)	2/8 (25%)
Caproic acid	H-CHO	1/8 (12.5%)	1/8 (12.5%)	3/8 (37.5%)	3/8 (37.5%)
Caproic acid	P-CPS	2/8 (25%)	0/8 (0%)	5/8 (62.5%)	1/8 (12.5%)

Table S9. All four active-condition-versus-PLA SCFA contrasts (post hoc).

The model includes participant and categorical period. β is the adjusted difference in log_2 post/pre change; 2^β is the ratio of post/pre fold changes. All intervals are unadjusted 95% t intervals. q_7 adjusts seven acids within a contrast, and q_{28} adjusts all 28 contrasts together. These comparisons do not isolate nutrient or erythritol effects. Figure 3 displays all estimates.

L-CHO versus PLA

Acid	β (95% CI)	2^β (95% CI)	P	q_7	q_{28}
Acetic acid	0.409 (-0.197 to 1.016)	1.33 (0.87–2.02)	0.1765	0.3088	0.2601
Propionic acid	0.613 (-0.710 to 1.937)	1.53 (0.61–3.83)	0.3485	0.4879	0.4470
Butyric acid	0.825 (-0.311 to 1.960)	1.77 (0.81–3.89)	0.1471	0.3088	0.2378
Isobutyric acid	1.490 (-0.255 to 3.235)	2.81 (0.84–9.42)	0.0907	0.3088	0.1954
Isovaleric acid	1.495 (-0.411 to 3.401)	2.82 (0.75–10.56)	0.1186	0.3088	0.2215
Valeric acid	0.813 (-1.439 to 3.066)	1.76 (0.37–8.37)	0.4633	0.5406	0.5406
Caproic acid	0.062 (-1.625 to 1.748)	1.04 (0.32–3.36)	0.9403	0.9403	0.9403

T-CPS versus PLA

Acid	β (95% CI)	2^β (95% CI)	P	q_7	q_{28}
Acetic acid	-0.088 (-0.678 to 0.502)	0.94 (0.62–1.42)	0.7605	0.7605	0.7887
Propionic acid	0.312 (-0.976 to 1.599)	1.24 (0.51–3.03)	0.6219	0.7605	0.6965
Butyric acid	0.196 (-0.909 to 1.301)	1.15 (0.53–2.46)	0.7173	0.7605	0.7725
Isobutyric acid	2.488 (0.790 to 4.186)	5.61 (1.73–18.20)	0.0059	0.0205	0.0334
Isovaleric acid	2.837 (0.982 to 4.691)	7.14 (1.98–25.84)	0.0043	0.0205	0.0334
Valeric acid	2.440 (0.248 to 4.632)	5.43 (1.19–24.79)	0.0306	0.0536	0.0952
Caproic acid	2.129 (0.488 to 3.770)	4.37 (1.40–13.64)	0.0132	0.0307	0.0526

H-CHO versus PLA

Acid	β (95% CI)	2^β (95% CI)	P	q_7	q_{28}
Acetic acid	0.415 (-0.165 to 0.996)	1.33 (0.89–1.99)	0.1529	0.1784	0.2378
Propionic acid	0.947 (-0.320 to 2.213)	1.93 (0.80–4.64)	0.1360	0.1784	0.2378
Butyric acid	0.501 (-0.586 to 1.588)	1.41 (0.67–3.01)	0.3512	0.3512	0.4470
Isobutyric acid	2.317 (0.646 to 3.987)	4.98 (1.57–15.85)	0.0086	0.0300	0.0400
Isovaleric acid	2.667 (0.842 to 4.491)	6.35 (1.79–22.49)	0.0060	0.0300	0.0334
Valeric acid	2.064 (-0.092 to 4.219)	4.18 (0.94–18.63)	0.0598	0.1396	0.1523
Caproic acid	1.316 (-0.298 to 2.930)	2.49 (0.81–7.62)	0.1053	0.1784	0.2106

P-CPS versus PLA

Acid	β (95% CI)	2^β (95% CI)	P	q_7	q_{28}
Acetic acid	0.219 (-0.386 to 0.825)	1.16 (0.77–1.77)	0.4621	0.4621	0.5406
Propionic acid	0.794 (-0.527 to 2.116)	1.73 (0.69–4.34)	0.2268	0.2646	0.3175
Butyric acid	0.987 (-0.147 to 2.122)	1.98 (0.90–4.35)	0.0850	0.1190	0.1954
Isobutyric acid	2.596 (0.853 to 4.338)	6.04 (1.81–20.23)	0.0052	0.0182	0.0334
Isovaleric acid	3.107 (1.203 to 5.011)	8.62 (2.30–32.24)	0.0025	0.0178	0.0334
Valeric acid	2.266 (0.016 to 4.515)	4.81 (1.01–22.86)	0.0485	0.0849	0.1358
Caproic acid	2.046 (0.362 to 3.730)	4.13 (1.29–13.27)	0.0193	0.0451	0.0677

Table S10. SCFA mass-composition and composite-endpoint analyses (post hoc).

The seven-acid CLR global test yielded pseudo-$F_{4,24}$=2.195, P=0.0199, and partial R^2=0.268. None of the seven CLR omnibus tests passed its BH family. Across 28 CLR contrasts, only T-CPS versus PLA propionate met q<0.05 (β=−0.805 natural-log CLR units, 95% CI −1.271 to −0.339; q=0.0437). All seven omnibus and 28 contrast rows are provided in the SCFA results CSV files in Supplementary Data S1, including nonsignificant findings.

Endpoint	F (4,24)	P	q (3 tests)	Partial R^2
Log_2 total SCFA	1.519	0.2283	0.2283	0.202
Branched mass percentage	3.311	0.0270	0.0437	0.356
Log_2 branched/major-straight ratio	3.245	0.0291	0.0437	0.351

Branched acids are isobutyrate+isovalerate. The mass percentage divides by the sum of all seven acids. The ratio denominator is acetate+propionate+butyrate. Percentages and ratios use mass units, not molar units.

Composite contrasts: joint BH adjustment across 12 comparisons.

Endpoint / condition vs PLA	β (95% CI)	P	q_{12}
Log_2 total SCFA / L-CHO	0.418 (-0.171 to 1.007)	0.1558	0.2078
Branched mass percentage / L-CHO	1.126 (-1.820 to 4.072)	0.4380	0.4778
Log_2 branched/major-straight ratio / L-CHO	1.057 (-0.745 to 2.859)	0.2378	0.2854
Log_2 total SCFA / T-CPS	0.097 (-0.476 to 0.671)	0.7288	0.7288
Branched mass percentage / T-CPS	4.605 (1.738 to 7.472)	0.0029	0.0282
Log_2 branched/major-straight ratio / T-CPS	2.648 (0.894 to 4.401)	0.0047	0.0282
Log_2 total SCFA / H-CHO	0.563 (-0.001 to 1.127)	0.0503	0.0987
Branched mass percentage / H-CHO	2.725 (-0.095 to 5.545)	0.0576	0.0987
Log_2 branched/major-straight ratio / H-CHO	1.960 (0.235 to 3.685)	0.0276	0.0663
Log_2 total SCFA / P-CPS	0.454 (-0.134 to 1.042)	0.1243	0.1864
Branched mass percentage / P-CPS	3.346 (0.404 to 6.288)	0.0275	0.0663
Log_2 branched/major-straight ratio / P-CPS	2.441 (0.641 to 4.240)	0.0100	0.0399

β is a percentage-point difference for branched mass percentage and a log_2-change difference for the other endpoints. Exponentiation gives ratios of fold changes only for the log_2 endpoints. The complete files also contain those ratios and within-contrast q values. No total-SCFA contrast passed q_{12}<0.05. P-CPS branched mass percentage had q_{12}=0.0663; its branched/major-straight ratio had q_{12}=0.0399. These findings remain exploratory.

Analytical-range sensitivity excluding caproic and valeric acids.

Five-acid profile	Pseudo-F	P	q (2 tests)	Partial R^2
Standardized concentration	1.694	0.0713	0.0713	0.220
Recomputed CLR composition	2.589	0.0296	0.0592	0.301

Both tests retained all 80 samples and 40 paired trials. These supplementary tests do not replace the seven-acid primary test or establish that common concentration effects have been excluded.

Table S11. Read counts, prevalence, and abundance for all 88 retained genus-level labels.

Counts use the unrarefied aggregate table and include zeros across 80 samples. The provider's fallback labels, including unresolved higher-rank placeholders, were retained when genus assignment was unavailable. IQR is reported as Q1–Q3. Mean relative abundance and prevalence are percentages. Complete taxonomy and filtering records are included in Supplementary Data S1. The median of label-wise median counts was 10.25 (range 0–3,476); 26 labels had a median ≤3 reads.

Delivered label	Median	Q1–Q3	Range	Prevalence %	Mean rel. %
Acinetobacter	9.00	0.00–14.00	0–29	71.25	0.0199
Actinomyces	33.50	11.00–61.25	0–461	91.25	0.1200
Adlercreutzia	119.00	5.25–406.25	0–2759	75.00	0.8761
Akkermansia	8.50	3.00–22.50	0–7572	78.75	0.5923
Alistipes	33.50	7.75–132.50	0–1415	86.25	0.4551
Allobaculum	6.50	0.00–15.00	0–42	65.00	0.0200
Anaerostipes	8.50	0.00–90.75	0–640	53.75	0.1667
Anaplasma	0.00	0.00–7.00	0–88	28.75	0.0210
Bacillus	0.00	0.00–4.50	0–31	27.50	0.0115
Bacteroides	886.50	182.75–2406.25	4–14276	100.00	4.3473
Barnesiella	0.00	0.00–25.00	0–329	42.50	0.0892
Bifidobacterium	2374.50	911.50–5744.50	183–18819	100.00	8.5361
Bilophila	0.00	0.00–6.00	0–57	47.50	0.0115
Blautia	3476.00	2269.75–4870.00	186–26273	100.00	9.7025
Bulleidia	2.00	0.00–7.00	0–100	52.50	0.0141
Butyricicoccus	28.00	7.00–65.50	0–1216	92.50	0.1280
Butyricimonas	0.50	0.00–35.50	0–168	50.00	0.0673
Clostridium	872.00	487.25–1880.50	99–9455	100.00	2.8667
Collinsella	2235.00	1199.00–3693.50	0–13321	93.75	6.3067
Coprococcus	1526.00	1050.00–2101.50	47–5807	100.00	4.2134
Desulfovibrio	0.00	0.00–8.25	0–255	47.50	0.0291
Dialister	198.50	10.75–807.00	0–8548	85.00	2.1028
Dorea	712.50	495.00–1307.00	118–3789	100.00	2.3218
Eggerthella	10.00	0.00–52.75	0–545	66.25	0.1124
Enterococcus	8.50	0.00–21.00	0–820	62.50	0.0881
Faecalibacterium	2515.50	1076.50–4710.75	2–10737	100.00	7.4422
Gemmiger	999.00	411.75–2305.75	0–10847	97.50	4.6128
Haemophilus	4.00	0.00–16.00	0–749	52.50	0.1263
Holdemania	0.00	0.00–7.25	0–228	46.25	0.0274
Lachnospira	23.00	8.00–51.25	0–290	82.50	0.1225
Lactobacillus	50.50	30.25–79.25	0–2871	96.25	0.2983
Lactococcus	0.00	0.00–4.00	0–160	32.50	0.0222
Leuconostoc	6.50	0.00–12.00	0–1473	65.00	0.0559
Megamonas	0.00	0.00–0.00	0–1963	20.00	0.0858
Megasphaera	0.00	0.00–0.50	0–1284	25.00	0.0555
Odoribacter	5.50	0.00–22.50	0–147	62.50	0.0460
Oscillospira	174.00	48.00–496.50	0–1703	97.50	0.8489
Parabacteroides	58.00	15.75–199.00	0–5655	91.25	0.5043
Paraprevotella	0.00	0.00–9.00	0–263	35.00	0.0265
Phascolarctobacterium	23.00	3.75–127.50	0–1826	83.75	0.3459
Prevotella	5.00	0.00–248.50	0–15928	62.50	1.9704
Pseudomonas	11.00	0.00–16.00	0–43	65.00	0.0249
Roseburia	1143.00	482.75–2728.25	24–8602	100.00	4.3677

Delivered label	Median	Q1–Q3	Range	Prevalence %	Mean rel. %
Rothia	6.50	0.00–20.00	0–136	55.00	0.0394
Ruminococcus	1050.00	167.00–1944.25	35–8268	100.00	3.6123
SMB53	80.00	26.75–177.25	0–2100	85.00	0.3169
Shigella	146.50	53.75–522.75	0–17845	96.25	1.7543
Slackia	0.00	0.00–4.50	0–242	25.00	0.0461
Streptococcus	443.50	163.00–1159.50	16–11590	100.00	2.2978
Subdoligranulum	32.00	9.50–73.25	0–120	91.25	0.1011
Sutterella	10.00	0.00–47.25	0–482	72.50	0.1284
Turicibacter	13.00	0.00–45.50	0–7981	70.00	0.4253
Veillonella	3.00	0.00–17.25	0–535	56.25	0.0456
Weissella	4.50	0.00–25.75	0–1886	55.00	0.2201
[Eubacterium]	44.50	5.75–392.00	0–2150	82.50	0.8345
[Ruminococcus]	761.50	528.75–1303.00	26–5683	100.00	2.5423
unclassified_Bacteria	32.00	13.75–53.25	0–427	97.50	0.1230
unclassified_Bacteroidales	0.50	0.00–6.25	0–62	50.00	0.0170
unclassified_Bifidobacteriaceae	7.00	0.00–21.25	0–116	71.25	0.0423
unclassified_Carnobacteriaceae	0.00	0.00–10.25	0–50	33.75	0.0147
unclassified_Clostridiales	251.00	167.50–309.25	36–847	100.00	0.6110
unclassified_Coriobacteriaceae	10.00	0.75–19.00	0–149	75.00	0.0455
unclassified_Enterobacteriaceae	59.00	20.75–320.00	0–14419	96.25	2.1318
unclassified_Erysipelotrichaceae	0.00	0.00–3.25	0–60	35.00	0.0110
unclassified_Firmicutes	4.00	1.00–11.00	0–52	77.50	0.0213
unclassified_Gemellaceae	0.00	0.00–8.00	0–56	41.25	0.0124
unclassified_Lachnospiraceae	271.50	176.00–437.00	33–1005	100.00	0.7797
unclassified_Lactobacillales	3.00	0.00–9.00	0–50	72.50	0.0147
unclassified_Pasteurellaceae	0.00	0.00–2.00	0–111	30.00	0.0120
unclassified_Peptostreptococcaceae	33.00	8.00–125.75	0–919	86.25	0.2624
unclassified_Rikenellaceae	10.50	0.00–53.50	0–916	70.00	0.1578
unclassified_Ruminococcaceae	1350.50	814.00–2590.50	130–12511	100.00	4.8622
unidentified_Bacteria	4.00	2.00–12.00	0–38	76.25	0.0193
unidentified_Bacteroidales	4.00	0.00–8.25	0–946	68.75	0.1568
unidentified_Christensenellaceae	0.00	0.00–10.00	0–315	40.00	0.0747
unidentified_Clostridiaceae	12.00	0.00–27.00	0–5089	66.25	0.2532
unidentified_Clostridiales	434.00	249.25–942.50	27–4195	100.00	1.6733
unidentified_Coriobacteriaceae	28.50	3.00–192.50	0–3855	83.75	0.5753
unidentified_Erysipelotrichaceae	440.00	158.00–1166.00	0–5199	97.50	1.8673
unidentified_Lachnospiraceae	1072.00	763.25–1545.25	233–5949	100.00	3.1089
unidentified_Peptostreptococcaceae	592.50	250.25–1784.50	11–8903	100.00	2.9255
unidentified_RF39	0.00	0.00–32.25	0–1302	37.50	0.1361
unidentified_Rikenellaceae	0.00	0.00–13.25	0–243	47.50	0.0330
unidentified_Ruminococcaceae	567.00	246.50–1002.25	4–5365	100.00	1.9029
unidentified_S24-7	2.00	0.00–7.00	0–27	53.75	0.0101
unidentified_TM7-3	1.50	0.00–6.25	0–70	51.25	0.0132
unidentified_[Barnesiellaceae]	0.00	0.00–2.00	0–262	30.00	0.0220
unidentified_[Mogibacteriaceae]	27.50	5.00–70.75	0–1060	80.00	0.1524

Pseudomonas and Acinetobacter had median read counts of 11 and 9, respectively. Their mean relative abundances were 0.0249% and 0.0199%. Omitting both labels and recomputing CLR yielded an 86-label global pseudo-$F_{4,24}$=0.992, P=0.4856, and partial R^2=0.142. This sensitivity does not identify contamination in the absence of appropriate controls.

Table S12. Two original-analysis chemical PC associations across annotation-exclusion scenarios.

Scenario	PC pair (metabolome–SCFA)	Partial ρ	P	q_{27}
All 1,051 features	PC1–PC1	0.653	0.0002	0.0054
All 1,051 features	PC2–PC2	0.538	0.0014	0.0189
Remove 2 matched SCFAs	PC1–PC1	0.659	0.0002	0.0054
Remove 2 matched SCFAs	PC2–PC2	0.541	0.0014	0.0189
Remove 12 related features	PC1–PC1	0.657	0.0002	0.0054
Remove 12 related features	PC2–PC2	0.542	0.0014	0.0189

All 27 PC correlations were repeated and BH adjusted together within each scenario. Only the two original-analysis associations are displayed here; the complete 81 correlations and all negative results are included in Supplementary Data S1. Original-analysis metabolome PC1 and PC2 explained 11.98% and 6.97% of variance. They explained 11.97% and 6.98% after the precise exclusion and 11.95% and 7.00% after broad exclusion. Remaining associations do not demonstrate causality or independent replication.

Table S13. Putative metabolomic annotations removed in the two sensitivity sets.

Provider ID / mode	Putative annotation	Formula	Set
M102T62 / pos	Isovaleric acid	$C_5H_{10}O_2$	Precise + broad
M115T139_2 / neg	Caproic acid	$C_6H_{12}O_2$	Precise + broad
M105T980 / pos	Hydroxypyruvic acid	$C_3H_4O_4$	Broad additional
M119T315 / pos	5-Hydroxypentanoic acid	$C_5H_{10}O_3$	Broad additional
M148T87 / pos	(R) 2,3-Dihydroxy-3-methylvalerate	$C_6H_{12}O_4$	Broad additional
M103T91 / neg	(R)-3-Hydroxybutyric acid	$C_4H_8O_3$	Broad additional
M105T79 / neg	Glyceric acid	$C_3H_6O_4$	Broad additional
M101T90 / neg	2-Ketobutyric acid	$C_4H_6O_3$	Broad additional
M101T988 / neg	Succinic acid semialdehyde	$C_4H_6O_3$	Broad additional
M129T283 / neg	4-Acetylbutyrate	$C_6H_{10}O_3$	Broad additional
M129T303 / neg	3-Methyl-2-oxovaleric acid	$C_6H_{10}O_3$	Broad additional
M130T283 / neg	Ketoleucine	$C_6H_{10}O_3$	Broad additional

The precise set contained two annotations, and the broad set contained these two plus ten additional annotations. Chemical rules are given in S1.6. The machine-readable manifest retains KEGG identifiers and feature-specific exclusion reasons. Annotation uncertainty remains unchanged by exclusion.

Table S14. Same-trial baseline-adjusted SCFA sensitivity (post hoc).

All eight participants and 40 trials were retained. Each next-morning endpoint was modeled with participant, categorical period, condition, and its same-trial pretrial value, on the same endpoint scale. Each model had one common baseline slope and 23 residual degrees of freedom. Confidence intervals are unadjusted 95% t intervals. These conditional next-morning effects are distinct from the original change-score effects in Tables S9 and S15.

(A) All 28 acid contrasts. Concentrations were $\log_2$ transformed; exponentiation gives a baseline-adjusted next-morning concentration ratio. BH q_{28} covers all four active conditions and seven acids. No contrast passed $q_{28}<0.05$.

L-CHO versus PLA

Acid	$\log_2$ difference (95% CI)	Adjusted ratio (95% CI)	P	q_{28}
Acetic acid	0.267 (-0.197–0.730)	1.20 (0.87–1.66)	0.2464	0.4058
Propionic acid	0.857 (-0.319–2.033)	1.81 (0.80–4.09)	0.1452	0.3682
Butyric acid	0.191 (-0.882–1.264)	1.14 (0.54–2.40)	0.7156	0.7707
Isobutyric acid	1.055 (-0.258–2.369)	2.08 (0.84–5.16)	0.1100	0.3682
Isovaleric acid	0.983 (-0.285–2.250)	1.98 (0.82–4.76)	0.1223	0.3682
Valeric acid	1.127 (-1.075–3.329)	2.18 (0.47–10.05)	0.3008	0.4211
Caproic acid	-0.048 (-1.672–1.575)	0.97 (0.31–2.98)	0.9515	0.9515

T-CPS versus PLA

Acid	$\log_2$ difference (95% CI)	Adjusted ratio (95% CI)	P	q_{28}
Acetic acid	0.159 (-0.302–0.620)	1.12 (0.81–1.54)	0.4826	0.5630
Propionic acid	0.607 (-0.544–1.759)	1.52 (0.69–3.38)	0.2864	0.4211
Butyric acid	-0.068 (-1.041–0.904)	0.95 (0.49–1.87)	0.8860	0.9188
Isobutyric acid	0.988 (-0.449–2.425)	1.98 (0.73–5.37)	0.1683	0.3682
Isovaleric acid	1.000 (-0.389–2.389)	2.00 (0.76–5.24)	0.1500	0.3682
Valeric acid	1.516 (-0.868–3.899)	2.86 (0.55–14.92)	0.2014	0.4027
Caproic acid	1.132 (-0.823–3.087)	2.19 (0.57–8.50)	0.2431	0.4058

Table S14. Baseline-adjusted acid contrasts (continued).

H-CHO versus PLA

Acid	Log_2 difference (95% CI)	Adjusted ratio (95% CI)	P	q_{28}
Acetic acid	0.332 (-0.109–0.773)	1.26 (0.93–1.71)	0.1329	0.3682
Propionic acid	0.898 (-0.215–2.012)	1.86 (0.86–4.03)	0.1087	0.3682
Butyric acid	0.465 (-0.475–1.406)	1.38 (0.72–2.65)	0.3169	0.4226
Isobutyric acid	1.228 (-0.110–2.566)	2.34 (0.93–5.92)	0.0702	0.3682
Isovaleric acid	1.348 (0.056–2.639)	2.55 (1.04–6.23)	0.0415	0.3682
Valeric acid	1.611 (-0.535–3.757)	3.05 (0.69–13.52)	0.1342	0.3682
Caproic acid	0.720 (-0.978–2.417)	1.65 (0.51–5.34)	0.3895	0.4957

P-CPS versus PLA

Acid	Log_2 difference (95% CI)	Adjusted ratio (95% CI)	P	q_{28}
Acetic acid	0.105 (-0.356–0.566)	1.08 (0.78–1.48)	0.6417	0.7187
Propionic acid	0.616 (-0.553–1.784)	1.53 (0.68–3.44)	0.2869	0.4211
Butyric acid	0.404 (-0.655–1.462)	1.32 (0.64–2.76)	0.4382	0.5334
Isobutyric acid	1.100 (-0.366–2.565)	2.14 (0.78–5.92)	0.1342	0.3682
Isovaleric acid	1.151 (-0.287–2.589)	2.22 (0.82–6.02)	0.1113	0.3682
Valeric acid	1.426 (-0.963–3.814)	2.69 (0.51–14.07)	0.2293	0.4058
Caproic acid	1.266 (-0.587–3.119)	2.41 (0.67–8.69)	0.1710	0.3682

Table S14. Baseline-adjusted composite endpoints (continued).

(B) All 12 composite contrasts. Log_2 total SCFA and log_2 branched-to-major-straight-chain ratio are exponentiated; branched mass percentage remains a difference in percentage points and is not exponentiated. BH q_{12} covers all three endpoints and four active-versus-PLA contrasts. No contrast passed $q_{12}<0.05$.

Log_2 total SCFA

Condition vs PLA	Difference (95% CI)	Adjusted ratio (95% CI)	P	q_{12}
L-CHO	0.305 (-0.149–0.760)	1.24 (0.90–1.69)	0.1779	0.2668
T-CPS	0.202 (-0.240–0.644)	1.15 (0.85–1.56)	0.3539	0.3686
H-CHO	0.478 (0.045–0.912)	1.39 (1.03–1.88)	0.0321	0.2614
P-CPS	0.249 (-0.212–0.711)	1.19 (0.86–1.64)	0.2748	0.3298

Branched mass percentage

Condition vs PLA	Difference (95% CI)	Adjusted ratio (95% CI)	P	q_{12}
L-CHO	1.167 (-1.466–3.800)	Not applicable	0.3686	0.3686
T-CPS	2.825 (-0.081–5.732)	Not applicable	0.0562	0.2614
H-CHO	1.961 (-0.627–4.549)	Not applicable	0.1307	0.2614
P-CPS	2.262 (-0.497–5.021)	Not applicable	0.1034	0.2614

Log_2 branched/major-straight ratio

Condition vs PLA	Difference (95% CI)	Adjusted ratio (95% CI)	P	q_{12}
L-CHO	0.792 (-0.462–2.047)	1.73 (0.73–4.13)	0.2043	0.2724
T-CPS	0.976 (-0.410–2.362)	1.97 (0.75–5.14)	0.1586	0.2668
H-CHO	0.960 (-0.301–2.221)	1.95 (0.81–4.66)	0.1289	0.2614
P-CPS	1.105 (-0.251–2.462)	2.15 (0.84–5.51)	0.1054	0.2614

All seven acid-specific and three composite omnibus tests, fitted baseline coefficients, numerical definitions, and complete contrast outputs are supplied in Supplementary Data S1. Omnibus tests use separate seven-acid and three-composite BH families. These post hoc models assess sensitivity to baseline handling; they do not establish equivalence, absence of an effect, or a cause of the baseline differences.

Table S15. Original P-CPS-versus-active-comparator SCFA contrasts on both scales.

These 21 original secondary comparisons complement the 28 active-versus-PLA comparisons in Table S9, giving 49 distinct reported change-score contrasts without repeating P-CPS versus PLA. Original estimates, unadjusted 95% intervals (24 residual degrees of freedom), P values, and q values are unchanged. Ratios are 2 raised to the $\log_2$ difference or confidence limit. q_7 adjusts seven acids separately within each original P-CPS comparison; it does not adjust all 49 comparisons together. No comparison below passed its original q_7<0.05 threshold.

P-CPS versus L-CHO

Acid	$\log_2$ change difference (95% CI)	Ratio of fold changes (95% CI)	P	q_7
Acetic acid	-0.190 (-0.807–0.427)	0.88 (0.57–1.34)	0.5314	0.7439
Propionic acid	0.181 (-1.166–1.528)	1.13 (0.45–2.88)	0.7836	0.7836
Butyric acid	0.163 (-0.993–1.319)	1.12 (0.50–2.49)	0.7738	0.7836
Isobutyric acid	1.105 (-0.671–2.881)	2.15 (0.63–7.37)	0.2112	0.3695
Isovaleric acid	1.612 (-0.328–3.552)	3.06 (0.80–11.73)	0.0992	0.3473
Valeric acid	1.452 (-0.840–3.745)	2.74 (0.56–13.40)	0.2035	0.3695
Caproic acid	1.984 (0.268–3.701)	3.96 (1.20–13.00)	0.0253	0.1769

P-CPS versus T-CPS

Acid	$\log_2$ change difference (95% CI)	Ratio of fold changes (95% CI)	P	q_7
Acetic acid	0.308 (-0.299–0.914)	1.24 (0.81–1.88)	0.3054	0.9198
Propionic acid	0.483 (-0.840–1.805)	1.40 (0.56–3.49)	0.4586	0.9198
Butyric acid	0.791 (-0.344–1.926)	1.73 (0.79–3.80)	0.1630	0.9198
Isobutyric acid	0.108 (-1.636–1.851)	1.08 (0.32–3.61)	0.8996	0.9198
Isovaleric acid	0.270 (-1.634–2.175)	1.21 (0.32–4.51)	0.7722	0.9198
Valeric acid	-0.174 (-2.425–2.076)	0.89 (0.19–4.22)	0.8743	0.9198
Caproic acid	-0.083 (-1.768–1.602)	0.94 (0.29–3.04)	0.9198	0.9198

P-CPS versus H-CHO

Acid	$\log_2$ change difference (95% CI)	Ratio of fold changes (95% CI)	P	q_7
Acetic acid	-0.196 (-0.802–0.410)	0.87 (0.57–1.33)	0.5112	0.8547
Propionic acid	-0.152 (-1.475–1.171)	0.90 (0.36–2.25)	0.8142	0.8547
Butyric acid	0.487 (-0.649–1.622)	1.40 (0.64–3.08)	0.3852	0.8547
Isobutyric acid	0.279 (-1.465–2.024)	1.21 (0.36–4.07)	0.7441	0.8547
Isovaleric acid	0.440 (-1.465–2.346)	1.36 (0.36–5.08)	0.6379	0.8547
Valeric acid	0.202 (-2.050–2.454)	1.15 (0.24–5.48)	0.8547	0.8547
Caproic acid	0.730 (-0.956–2.416)	1.66 (0.52–5.34)	0.3805	0.8547

Figure S1. Absolute fecal SCFA concentrations summarized by condition and time.

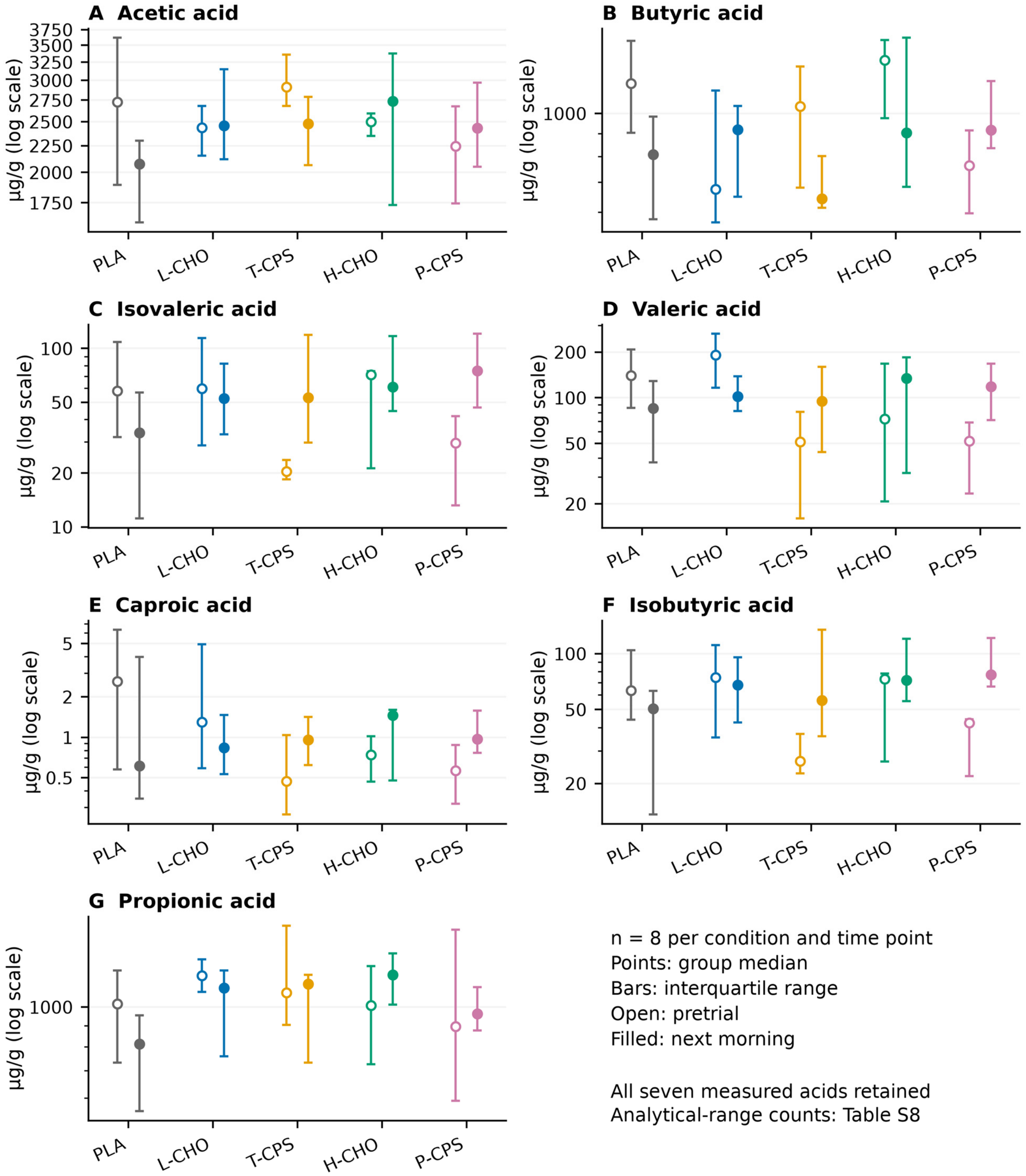

Points show group medians and bars the interquartile range (n=8 per condition/time). Open circles denote pretrial values and filled circles next-morning values. Concentrations are µg/g of fecal sample as weighed, with logarithmic axes. All provider-reported positive estimates are retained; analytical-range counts are reported in Table S8. No individual paired observations are displayed.

Figure S2. SCFA mass composition, total concentrations, and branched-acid percentage.

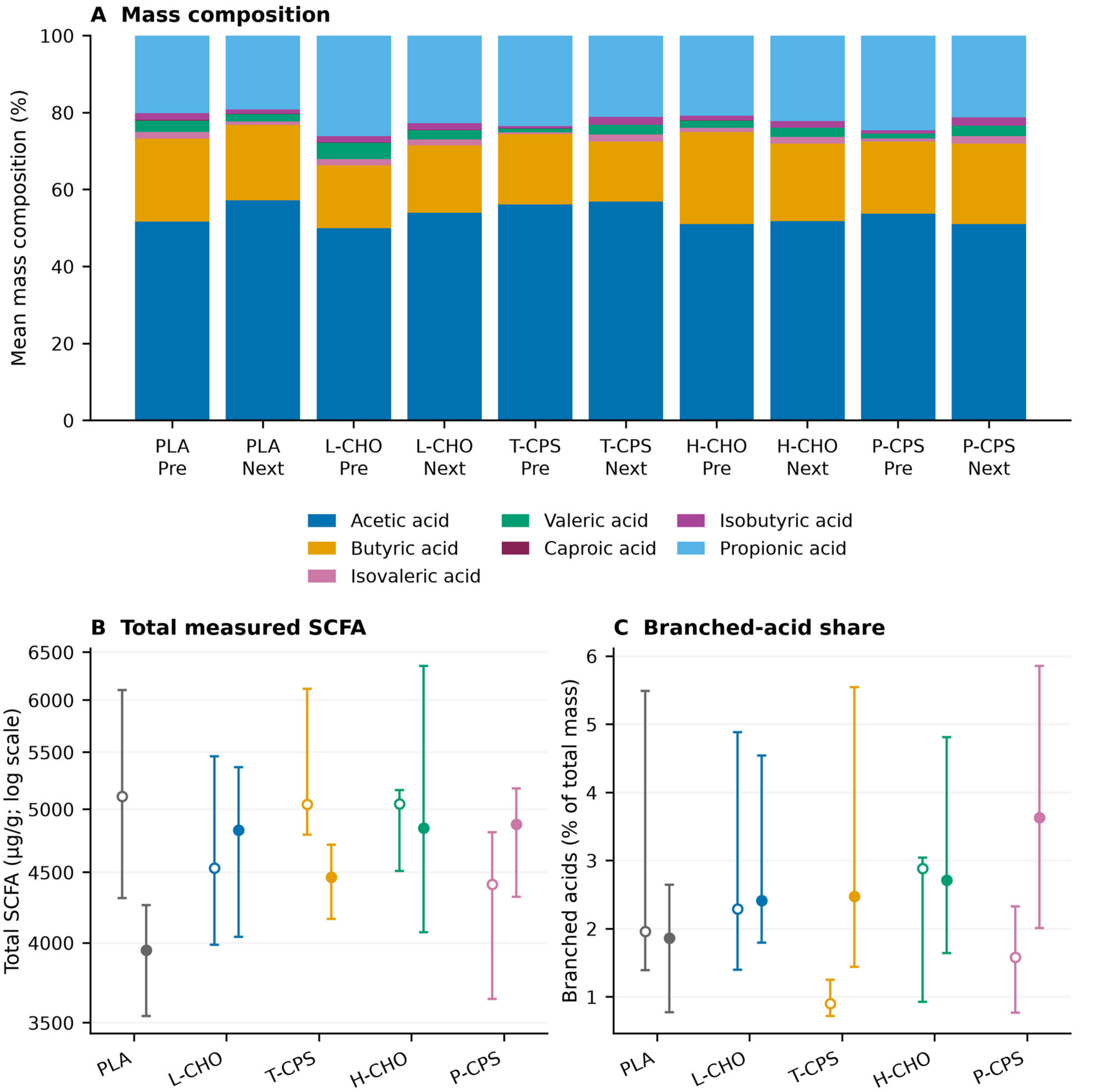

(A) Arithmetic means of sample-wise mass fractions across the seven acids (n=8 per condition/time). (B) Median and interquartile range of total measured SCFA concentrations (µg/g; logarithmic axis). (C) Median and interquartile range of isobutyrate+isovalerate as a percentage of total measured SCFA mass. In panels B and C, open circles denote pretrial values and filled circles next-morning values. These are mass proportions, not molar fractions. All provider-reported estimates are retained; analytical-range flags and complete statistical families are reported separately. No individual observations are displayed.

Table S16. Completed rowing distance by condition in the multi-omics subset and parent cohort.

Condition	Multi-omics subset (n=8) Mean (SD), m	Parent cohort (n=12) Mean (SD), m
PLA	10415.8 (629.2)	10643.9 (675.3)
L-CHO	11263.4 (1078.4)	11334.0 (905.4)
T-CPS	11506.9 (1052.4)	11563.8 (897.2)
H-CHO	11424.4 (903.5)	11685.0 (880.9)
P-CPS	12150.3 (738.5)	12387.7 (785.7)

Distances sum the two 30-min bouts. The eight participants are a subset of the 12 completers; these columns are not independent cohorts. Each condition contains one trial per included participant. Values are descriptive and do not introduce performance-efficacy testing. See Supplementary Methods S1.11.

Table S17. SILVA 138.2 taxonomic sensitivity analyses (post hoc).

Analysis	Labels	Pseudo-$F_{4,24}$	P	Partial R^2	Global q
Original annotations	88	1.067	0.2813	0.151	0.2813
SILVA 138.2	130	1.110	0.1932	0.156	0.1932

The original row reports the original primary microbiome test. The SILVA row belongs to the alternative three-test family, which yielded metabolome q=0.1334, SCFA q=0.0963, and microbiome q=0.1932. The SILVA null partial R^2 median was 0.143 and its 95th percentile was 0.169. Classification and aggregation rules are given in S1.9.

(A) Label-level omnibus tests passing BH correction across all 130 labels.

Label	$F_{4,24}$	P	q
Lactiplantibacillus	19.541	2.88e-07	3.74e-05
Lactobacillus	7.443	0.000478	0.0207
Pseudomonas	9.079	0.00013	0.0084

Median read counts across all 80 specimens, including zeros, were 0 for Lactiplantibacillus, 12 for Pseudomonas, and 9 for Lactobacillus. These low-count, annotation-sensitive signals do not imply absolute bacterial growth, benefit, or causality. All 130 omnibus results are supplied in Supplementary Data S1.

Table S17 (continued). P-CPS contrasts passing their separate 130-label BH families.

Label	Comparator	CLR difference (95% CI)	P	q
Lactiplantibacillus	PLA	-4.117 (-5.730, -2.505)	2.1e-05	0.0027
Lactobacillus	PLA	-2.782 (-3.979, -1.584)	7.04e-05	0.0046
Lactobacillus	L-CHO	-2.604 (-3.825, -1.383)	0.00019	0.0203
Ligilactobacillus	L-CHO	-3.372 (-5.026, -1.717)	0.000313	0.0203
Ligilactobacillus	T-CPS	-3.102 (-4.726, -1.478)	0.00061	0.0265
Acinetobacter	T-CPS	-3.722 (-5.581, -1.863)	0.000377	0.0245
Pseudomonas	T-CPS	-4.162 (-5.604, -2.721)	3.77e-06	0.000491
Lactiplantibacillus	H-CHO	-3.627 (-5.241, -2.012)	0.000105	0.0136
Lactobacillus	H-CHO	-2.351 (-3.550, -1.152)	0.000468	0.0304

Differences are P-CPS minus the named comparator in next-morning minus pretrial natural-log CLR change, adjusted for participant and categorical period. Intervals are unadjusted 95% t intervals with 24 residual degrees of freedom. BH q adjusts 130 labels separately within each of the four contrast families; it does not adjust all 520 tests jointly or correct across alternative analytical pipelines. All 520 contrasts, including nonsignificant results, are supplied in Supplementary Data S1.

Table S18. Retained untargeted-metabolomics annotation evidence.

Evidence item	Result
Features	1,051 (720 positive mode; 331 negative mode)
Provider library field	BioDeepDB 732; MetaDNA 137; MoNA 100; mzCloud 79; GNPS 3
Provider “level 2” code	1,051 entries; not independently verified MSI assignments
Matching identification plots	1,051; plot availability is not verified acquired-MS2 coverage
Inspected plots	Six examples spanning all five library labels displayed experimental/reference mirror spectra
Feature-specific acquisition flags or numerical match scores	Not present in the delivered annotation tables
Verified acquired-MS2 coverage	Not determinable from the retained records
Absolute annotation error >15 ppm	175 of 1,051 entries
Absolute annotation error >5 ppm	397 of 1,051 entries
Maximum absolute annotation error	29.9177 ppm

The ppm audit concerns the delivered annotation-matching field, not calibration accuracy. The <30 ppm annotation window exceeds the 15 ppm peak-detection tolerance and the instrument’s routine specification under defined conditions. Names remain provisional candidate labels; a blanket MSI level-2 assignment is not made. Class-level MSI level 3 requires supporting class evidence; otherwise the identity remains unknown (MSI level 4). Identification plots cannot replace feature-to-scan mapping, documented match quality, authentic-standard confirmation, or unavailable acquisition files. See manuscript Section 2.6 and Supplementary Methods S1.10.
