# Supplementary material for: Fecal Metabolic and Microbial Responses to Five Acute Fueling Strategies During a 60-Minute Rowing Protocol: A Randomized Crossover Multi-Omics Pilot Study
Source: Metabolites. 2026 Sep 28;16(10):718. doi: 10.3390/metabo16100718 (PMC13707109; doi:10.3390/metabo16100718)
Supplement: Supplementary file 1 [file metabolites-16-00718-s001.zip › metabolites-4529093_Rev2_SupplementaryDataS1_Code_and_Summary_Results.zip!/figures.zip!/Figure2_aggregate_PCA.pdf]

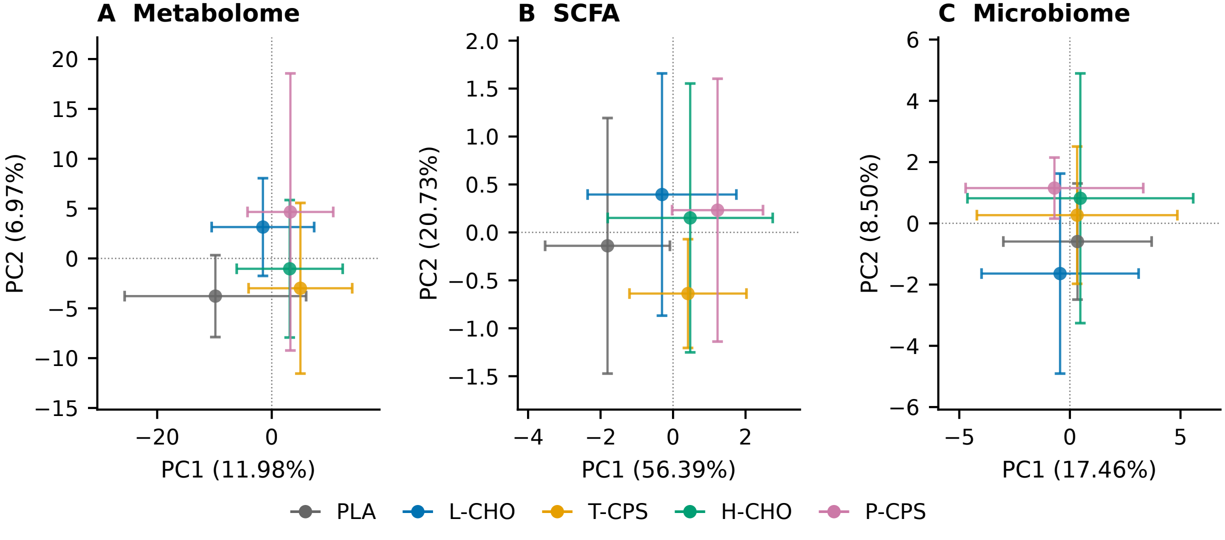

Condition means ± sample SD; n = 8 paired trials per condition
