# Supplementary material for: Fecal Metabolic and Microbial Responses to Five Acute Fueling Strategies During a 60-Minute Rowing Protocol: A Randomized Crossover Multi-Omics Pilot Study
Source: Metabolites. 2026 Sep 28;16(10):718. doi: 10.3390/metabo16100718 (PMC13707109; doi:10.3390/metabo16100718)
Supplement: Supplementary file 1 [file metabolites-16-00718-s001.zip › metabolites-4529093_Rev2_SupplementaryDataS1_Code_and_Summary_Results.zip!/figures.zip!/Figure4_aggregate_PC_correlations.pdf]

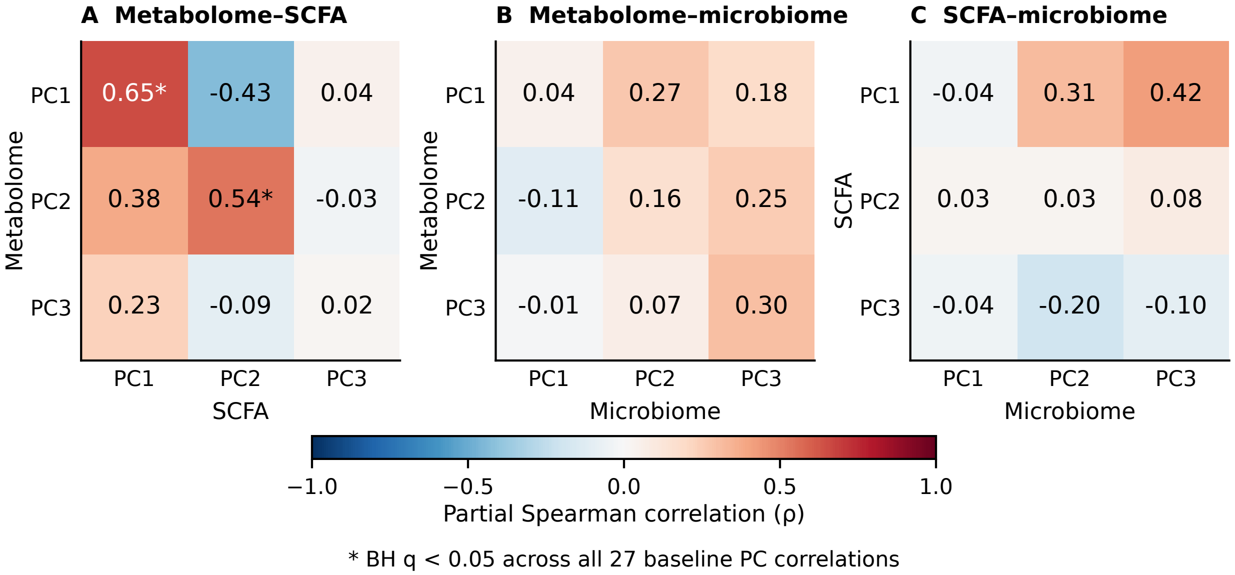
A Metabolome–SCFA
B Metabolome–microbiome
C SCFA–microbiome
Metabolome
SCFA
Microbiome
PC1
PC2
PC3
0.65*
-0.43
0.04
0.38
0.54*
-0.03
0.23
-0.09
0.02
0.04
0.27
0.18
-0.11
0.16
0.25
-0.01
0.07
0.30
-0.04
0.31
0.42
0.03
0.03
0.08
-0.04
-0.20
-0.10
−1.0
−0.5
0.0
0.5
1.0
Partial Spearman correlation (ρ)
* BH q < 0.05 across all 27 baseline PC correlations
