# Supplementary material for: Fecal Metabolic and Microbial Responses to Five Acute Fueling Strategies During a 60-Minute Rowing Protocol: A Randomized Crossover Multi-Omics Pilot Study
Source: Metabolites. 2026 Sep 28;16(10):718. doi: 10.3390/metabo16100718 (PMC13707109; doi:10.3390/metabo16100718)
Supplement: Supplementary file 1 [file metabolites-16-00718-s001.zip › metabolites-4529093_Rev2_SupplementaryDataS1_Code_and_Summary_Results.zip!/figures.zip!/Figure_S2_SCFA_composition_summary.pdf]

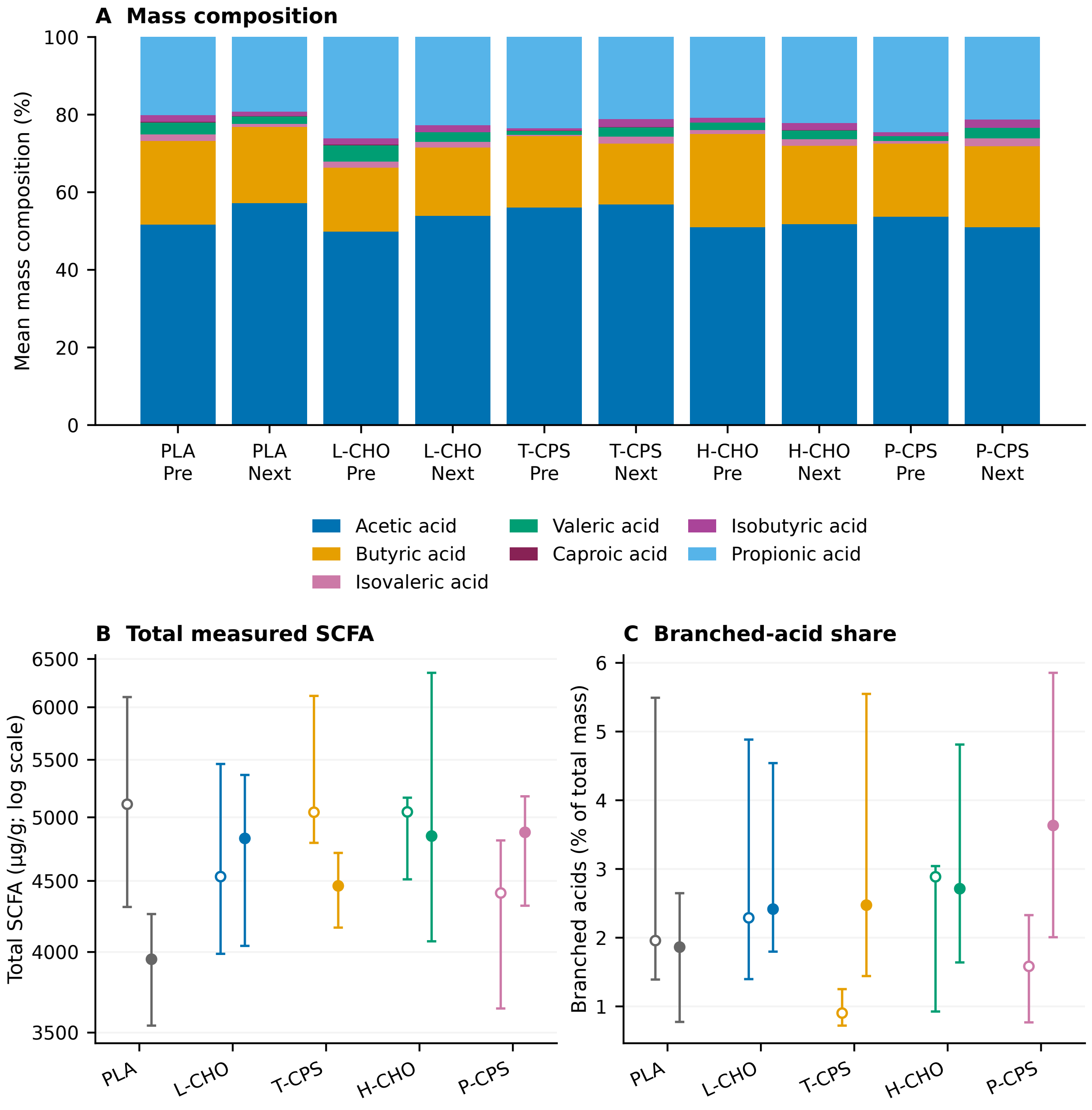

A Mass composition
Mean mass composition (%)
100
80
60
40
20
0
PLA Pre
PLA Next
L-CHO Pre
L-CHO Next
T-CPS Pre
T-CPS Next
H-CHO Pre
H-CHO Next
P-CPS Pre
P-CPS Next
Acetic acid
Valeric acid
Isobutyric acid
Butyric acid
Caproic acid
Propionic acid
Isovaleric acid
B Total measured SCFA
Total SCFA (µg/g; log scale)
6500
6000
5500
5000
4500
4000
3500
PLA
L-CHO
T-CPS
H-CHO
P-CPS
C Branched-acid share
Branched acids (% of total mass)
6
5
4
3
2
1
PLA
L-CHO
T-CPS
H-CHO
P-CPS
